# Enhanced and stabilized hydrogen production from methanol by ultrasmall Ni nanoclusters immobilized on defect-rich h-BN nanosheets

**DOI:** 10.1073/pnas.2015897117

**Published:** 2020-11-09

**Authors:** Zhuolei Zhang, Ji Su, Ana Sanz Matias, Madeleine Gordon, Yi-Sheng Liu, Jinghua Guo, Chengyu Song, Chaochao Dun, David Prendergast, Gabor A. Somorjai, Jeffrey J. Urban

**Affiliations:** ^a^The Molecular Foundry, Lawrence Berkeley National Laboratory, Berkeley, CA 94720;; ^b^Materials Sciences Division, Lawrence Berkeley National Laboratory, Berkeley, CA 94720;; ^c^Applied Science and Technology Graduate Group, University of California, Berkeley, CA 94720;; ^d^Advanced Light Source, Lawrence Berkeley National Laboratory, Berkeley, CA 94720;; ^e^The National Center for Electron Microscopy, Lawrence Berkeley National Laboratory, Berkeley, CA 94720;; ^f^Department of Chemistry, University of California, Berkeley, CA 94720

**Keywords:** LOHCs, methanol dehydrogenation, metal–support interaction, transition metal, BN

## Abstract

Employing liquid organic hydrogen carriers (LOHCs) to transport hydrogen to where it can be utilized relies on methods of efficient chemical dehydrogenation to access this fuel. Therefore, developing effective strategies to optimize the catalytic performance of cheap transition metal-based catalysts in terms of activity and stability for dehydrogenation of LOHCs is a critical challenge. Here, we report the design and synthesis of ultrasmall nickel nanoclusters (∼1.5 nm) deposited on defect-rich boron nitride (BN) nanosheet (Ni/BN) catalysts with higher methanol dehydrogenation activity and selectivity, and greater stability than that of some other transition-metal based catalysts. The interface of the two-dimensional (2D) BN with the metal nanoparticles plays a strong role both in guiding the nucleation and growth of the catalytically active ultrasmall Ni nanoclusters, and further in stabilizing these nanoscale Ni catalysts against poisoning by interactions with the BN substrate. We provide detailed spectroscopy characterizations and density functional theory (DFT) calculations to reveal the origin of the high productivity, high selectivity, and high durability exhibited with the Ni/BN nanocatalyst and elucidate its correlation with nanocluster size and support–nanocluster interactions. This study provides insight into the role that the support material can have both regarding the size control of nanoclusters through immobilization during the nanocluster formation and also during the active catalytic process; this twofold set of insights is significant in advancing the understanding the bottom-up design of high-performance, durable catalytic systems for various catalysis needs.

## Significance

While transition metal-based catalysts promise lower-cost alternatives to traditional precious metals, their low activity and stability limit their deployment within industrial dehydrogenation. Here, we report the design and synthesis of ultrasmall nickel nanoclusters (∼1.5 nm) deposited on defect-rich BN nanosheet (Ni/BN) catalysts with excellent methanol dehydrogenation activity and selectivity. We found an idiosyncratic metal–support interaction not only plays a vital role in promoting the one-pot synthesis of ultrasmall Ni nanoclusters with high catalytic activity, helping to disperse and anchor the nanoclusters but also strongly enhancing the resistance to sintering and coking during methanol dehydrogenation. Calculated turnover frequency (TOF) is among the best compared with some other dehydrogenation catalysts reported previously.

Driven by the environmental consequences of fossil energy consumption, hydrogen, a potentially renewable and sustainable resource for clean energy, has gradually evolved into an energetic area of research globally (1–3). In the hydrogen landscape, one of the key links bridging the gap between sustainable production and utilization is hydrogen storage and transportation (4, 5). In contrast to the conventional storage and transportation approaches of compressed gas, or solid-state storage, liquid organic hydrogen carriers (LOHCs), such as methanol (6, 7), have attracted significant attention. LOHCs are appealing due to their high gravimetric hydrogen content, relatively low cost, easy handling and transportation, as well as their ability to be manufactured from a variety of renewable sources (8–10). Based on this point, selective dehydrogenation of methanol by heterogeneous catalysts plays a vital role not only in fundamental research but also in practical industries. Currently, the noble-metal–based catalysts exhibit a high activity but are hindered by exorbitant pricing, low abundance, and susceptibility to CO poisoning (11, 12).Transition metal-based catalysts, by contrast, promise lower cost, but low activity and stability levels (mainly due to coke deposition and particle sintering) limit their potential deployment into dehydrogenation industries. Despite great efforts, developing effective strategies to greatly optimize the catalytic performance of cheaper transition metal-based catalysts, such as nickel-based catalysts for dehydrogenation of LOHCs, still remains a great, ongoing challenge (13, 14).

Effective strategies to tune the catalytic properties of the transition metal-based catalysts is to take advantage of the size effect and metal–support interaction effect (15–19). From the viewpoint of reactivity, ultrasmall nanoclusters are highly preferred as the reduction of the size significantly increases the amount of surface sites per unit weight and is a reliable method to produce more active catalysts. Thus, one of the most important objectives is to construct nanoclusters that are both small and monodisperse in size. However, the instability and aggregation of small nanocluster (sintering) affect their catalytic activity, consequently limiting the use for industrial applications. To overcome these drawbacks, design of a proper catalyst support was employed to suppress metal sintering and tremendously affect the catalytic properties of catalysts. The supports could not only help to disperse and anchor the nanoclusters, they also tremendously facilitate catalytic action and heighten overall performance by means of interacting with the metal nanoclusters (20–27).

By virtue of an exceptional chemical and thermal stability, large surface area, high thermal conductivity, and strong surface adsorbing capability, two-dimensional (2D) hexagonal BN nanosheets (h-BN or BN in this paper), a structural analog of graphene, are one of the most attractive transition metal catalysts supports (28–30). The pristine BN surface is an inert support for metal nanoclusters, which would lead to catalyst deactivation by sintered metal species (31, 32). Thanks to the chemical modification/doping or defect engineering, BN owns greater possibilities in changing physical and chemical characteristics for catalysis development. However, the role of support in practical catalysts is still not clearly understood on various occasions as it is rather more complex (33). The size control of transition metal such as Ni is well known to be very sensitive to the preparation methods. Revealing the role of the support during the nanocluster formation and deposition is a prerequisite, and it may provide clues to an understanding of their role in subsequent catalytic processes.

Therefore, here in this paper, we present surface-modified BN nanosheets with abundant O-terminated vacancies that not only play a vital role in promoting the one-pot synthesis of ultrasmall Ni nanoclusters (∼1.5 nm) with high catalytic activity, helping to disperse and anchor the nanoclusters, but that also strongly interact with these nanoclusters to enhance the sintering resistance and coke inhibition properties during the methanol dehydrogenation. We provide detailed spectroscopy characterizations and density functional theory (DFT) calculations to monitor the transformation of the BN nanosheets, which clarified the role of the substrate during the ultrasmall Ni nanoclusters formation and deposition. We found the Ni nanoclusters nucleation preferably takes place at the BN_2_O defects. A “pit” model structure was thus suggested for the Ni/BN system, in which the nanoclusters occupy pristine regions of the BN nanoflakes and interact with nearby BN edges, preserving the ultrasmall size of the nanoparticles. This facilitates their catalytic action in the process of methanol adsorption, CO and H_2_ transferring, endowing the catalysts with excellent selectivity, productivity, and stability performance. Besides, calculated turnover frequency (TOF) was found among the best compared with some other transition metal-based catalysts reported previously. Our discovery gives an understanding of the interfacial interaction between Ni nanoclusters and defect-rich BN nanosheet support both in the nanoclusters formation and catalytic process, which may enlighten the path of optimizing LOHC dehydrogenation research in the perspective of supported catalysts.

## Results and Discussion

### Synthesis and Structure Characterizations.

To optimize the catalytic performance of the BN support, exfoliation to increase surface area appears to be the obvious route. However, the partial ionic character in the B–N bond and the “lip–lip” interactions between neighboring layers make exfoliating BN more challenging than with other kinds of layered materials (34–36). Despite numerous reports discussing the exfoliation of BN, few of these methods can meet the demand of producing large surface area sheets with high yield and purity (37). To satisfy these requirements, here we used a two-step gas expansion and alkali intercalation approach to effectively produce large amounts of several layered boron nitride nanosheets with abundant vacancies.

**Fig. 1.**
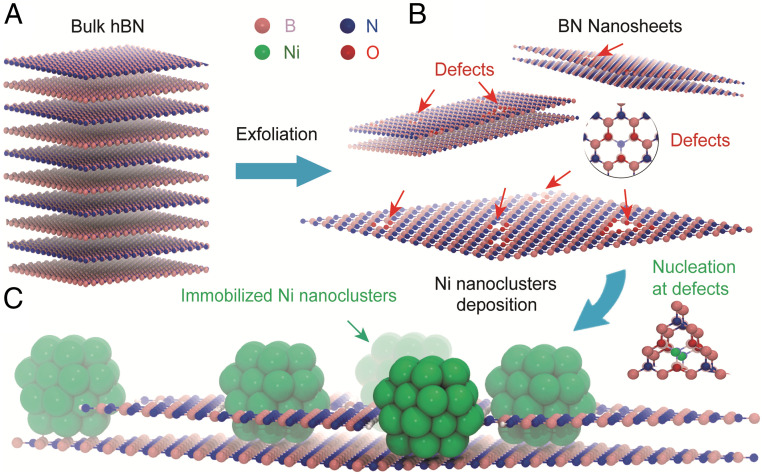
Schematic illustration of the formation of defective BN nanosheets and further deposition of Ni nanoclusters. Defective BN nanosheets (*B*) were prepared from bulk BN (*A*) by a combined exfoliation method of gas exfoliation and lithium intercalation-based exfoliation. Ni nanoclusters were deposited by in situ reaction of lithium naphthalenide with [Ni(Cp)_2_] (Cp = C_5_H_5_) in the BN nanosheets solution. Proposed “pit” structure of the Ni/BN nanocomposite (*C*).

Fig. 1 shows the schematic illustration of the processing steps in obtaining Ni/BN nanocomposite where ultrasmall nickel nanoclusters were deposited on the vacancy-abundant BN nanosheets. First, bulk BN powders are thermally expanded at a high temperature (800 °C, lower than the oxidation temperature) and pre-exfoliated by rapidly immersion in liquid nitrogen (38). The instant gasified liquid N_2_ works well in broadening BN interspace exceedingly due to the significant role played by high temperatures. Thermal fluctuations provide transient lattice openings enabling liquid N_2_ to diffuse among each interlayer in BN. Specifically, the interaction between the neighboring layer wanes at high temperature, while simultaneously the interlayer distance is increased (38). This combined effect results in extreme temperature gradients in the BN, from which the curling and delamination actions benefit. In the second step, the expanded BN nanosheets were dispersed in lithium-naphthalenide (LiNaph) solution in tetrahydrofuran (THF), followed by sonication (70 °C) to further be exfoliated into few-layered BN nanosheets. LiNaph is formed by reacting lithium metal with naphthalene, which was found to be an effective reagent for exfoliating 2D nanomaterials. The metal transfers an electron to the aromatic system to form a radical anion, facilitating the ion-electron transfer topotactic reaction for the exfoliation (39–41). A dark green solution consisting predominantly of few-layered BN can be obtained after centrifugation and decanting the supernatant. We found clear oxidation peaks observed in X-ray absorption spectroscopy (XAS) at the B K-edge of BN during the exfoliation (see Fig. 4*A*; will discuss in detail later). This probably attributes to the hygroscopic nature of THF that has rich chemistry involving autooxidation of THF in the presence of O_2_ and H_2_O under sonication, leading to the formation of active intermediate species, such as radicals and hydroperoxides, and may further oxidize BN edges and facilitate nanosheet exfoliation from it (42–44). The merit to having the LiNaph in the system is its relatively high reducing potential which aids in the deposition of ultrasmall Ni nanoclusters on BN nanosheets [*E*_0_([LiNaph]/THF) = −3.1 V] (45–47). In the third step, the nickel nanoclusters were prepared by an in situ reaction of LiNaph with [Ni(Cp)_2_] (Cp = C_5_H_5_) in the BN nanosheets solution. According to the model given by LaMer, the high reducing capability causes rapid nucleation of metal atoms. Thus, the high oversaturation of metal atoms results in such rapid nucleation that ultrasmall nanoclusters are formed when an equilibrium state is reached (48, 49). Compared to the nickel nanoclusters prepared by other methods with various reducing agents reported previously, the nanoclusters we prepared (∼1.5 nm) are among the smallest (50–52). Subsequent to acetonitrile washing, centrifugation, and high-temperature vacuum drying, the Ni/BN nanocomposite can be obtained as light blue-gray powder samples for further use.

**Fig. 2.**
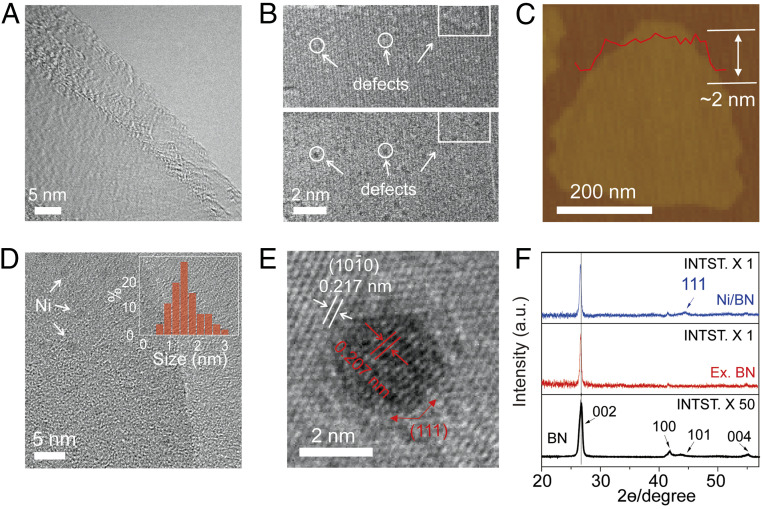
Morphology and structure characteristics of exfoliated BN nanosheets and Ni/BN nanocomposite. (*A*) Low-resolution transmission electron microscopy (TEM) image of typical exfoliated BN nanosheets. (*B*) Aberration-corrected high-resolution TEM (HRTEM) images of typical exfoliated BN nanosheet at the same location at different focus (*Top*, under focus; *Bottom*, over focus). (*C*) Atomic force microscopy (AFM) image of a typical exfoliated BN nanosheet. (*D*) The low-resolution TEM image of typical Ni nanoclusters deposited on exfoliated BN nanosheets. (*Inset*) Size distribution of Ni nanoclusters. (*E*) HRTEM images of a typical Ni nanocluster deposited on exfoliated BN nanosheets, which clearly shows the lattice structure of BN nanosheet and Ni nanocluster, respectively. (*F*) X-ray powder diffraction (XRD) patterns of bulk BN, exfoliated BN nanosheets, and Ni/BN nanocomposite.

To illustrate the morphological change after exfoliation, transmission electron microscopy (TEM) images were recorded on bulk BN powder (*SI Appendix*, Fig. S1) and exfoliated few-layered BN nanosheets (Fig. 2*A*). The bulk BN powder exhibits irregular flake shapes with the thickness in the range of tens of nanometers, while the exfoliated BN nanosheets show ultrathin, transparent morphologies with wrinkle structures distributed across the thin film, indicating the thorough exfoliation of pristine BN into a thin film structure. From high-resolution spherical aberration-corrected TEM image in Fig. 2*B*, abundant defects were observed. We recorded a series of images at different focuses with focal steps of 2 nm (*SI Appendix*, Fig. S2), where the white dots (representing the point defects, *Top* image of Fig. 2*B*) gradually turns to black dots (*Bottom* image of Fig. 2*B*), indicating the missing atoms at these locations. Furthermore, atomic force microscopy (AFM) images were recorded on a typical BN nanosheet (Fig. 2*C*), exhibiting a linescan profile thickness of roughly 2 nm. The percentage of monolayer BN nanosheets in our products was around 10%, and most thicknesses fell in a range of 1 to 3 nm. Fig. 2*D* shows the TEM image of the Ni/BN nanocomposite with the nickel nanoclusters of about 1.5 nm uniformly distributing on the BN nanosheets. The supported BN nanosheets show the lattice distance of about 0.217 nm, which corresponds to (1010) crystal planes of hexagonal BN. As a comparison, the deposited Ni clusters were crystalline (Fig. 2*E*) with a lattice distance of 0.207 nm, corresponding to the (111) crystal planes of fcc Ni.

X-ray powder diffraction (XRD) was performed to further investigate the crystalline structure. The comparative XRD patterns are present in Fig. 2*F*, where the exfoliated sample exhibits considerable changes comparing to its bulk counterpart. The bulk BN has strong XRD peaks, which were indexed to be (002), (100), (101), and (004) according to JCPDS card no. 85-1068. The intensity of these peaks was significantly decreased to nearly 2% after exfoliation (*SI Appendix*, Fig. S3). Besides, the (002) peak slightly downshifts from 26.75° to 26.65° after exfoliation, indicating the increased interplanar distance. The interplanar distance increase as well as the diffraction peaks intensity decrease confirms the lower level stacking of h-BN nanosheets along the c direction (53, 54). Furthermore, when deposited with Ni nanoclusters, a new peak appeared at 44.48°, corresponding to the (111) crystal planes of fcc Ni. The slight downshifts of the (002) peak to 26.57° indicates that the in situ deposition of nanoclusters may have the capability to increase the interplanar distance of BN nanosheets during the nanoclusters’ growth to further help the exfoliation process.

### Spectroscopic Characterizations.

**Fig. 3.**
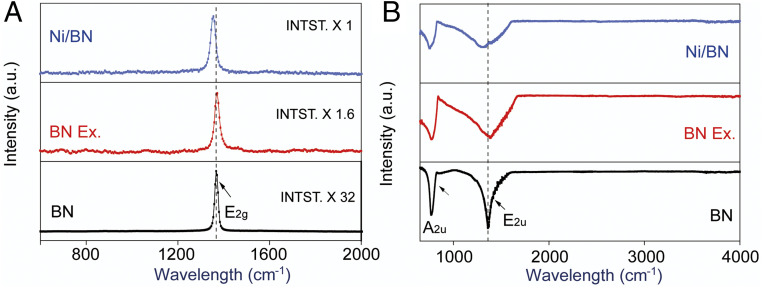
Spectroscopic characteristics of pristine bulk BN, exfoliated BN nanosheets, and Ni/BN nanocomposite. (*A*) The Raman spectra of typical pristine bulk BN, exfoliated BN nanosheets, and Ni/BN nanocomposite. (*B*) Fourier-transform infrared spectroscopy (FTIR) spectra of pristine BN, BN nanosheets, and Ni/BN nanocomposite.

Raman scattering is known to be a sensitive tool to detect the disorder induced by impurities and defects, which have been demonstrated to be very effective in studying graphene-like materials (55, 56). The Raman spectra are presented in Fig. 3*A*. The strong peak at 1,367.1 cm^−1^ represents the high-frequency interlayer Raman active E_2g_ mode. Compared to the bulk BN, BN nanosheets and Ni/BN nanocomposite reveal a much lower peak intensity resulting from the reduction of layer thickness and probably the creation of defects during the exfoliation. Furthermore, the G band blue shifts to 1,371.2 cm^−1^ after exfoliation and then red shifts to 1,356.7 cm^−1^ when deposited with Ni nanoclusters. The reduction of the h-BN layer numbers causes the blue shift and, at the same time, leads to the in-plane strain strengthening and interlayer interaction weakening; whereas the red shift indicates the reduced in-plane strain due to the localized fixation effect by the deposited nanoclusters (57). In addition, the full width at half-maximum (FWHM) of the G band was found to be broadened after exfoliation from 17.9 to 25.4 cm^−1^. The association of G band and the vibration of the hexagonal ring in-plane B–N relative motion, presumably indicates that the FWHM swelling can be ascribed to the generation of new defects in the nanosheet surface. Fig. 3*B* shows the Fourier transform infrared spectroscopy (FTIR) spectra of pristine BN, BN nanosheets, and Ni/BN nanocomposite. The bulk BN E_2u_ characteristic peak resulting from the in-plane B–N stretching is observed at 1,368.4 cm^−1^ in the figure, and its A_2u_ peak representing the out-of-plane B–N bending vibration is locating at 771.6 cm^−1^ (58). A blue shift appears after exfoliation to 1,392.4 and 773.3 cm^−1^ of both of the two aforementioned peaks, respectively, arising from the intensification of both of the B–N bonds stretching, and especially bending vibration caused by the BN thinning behavior. Moreover, a red shift of the two peaks to 1,336.4 and 763.5 cm^−1^ occurs during the Ni nanoclusters deposition, due to the localized fixation effect by the deposited nanoclusters. Like the characteristics of the observed Raman peaks, the decreased peak intensity and the broadened peak FWHM can probably be attributed to the newly generated defects at the nanosheet surface.

**Fig. 4.**
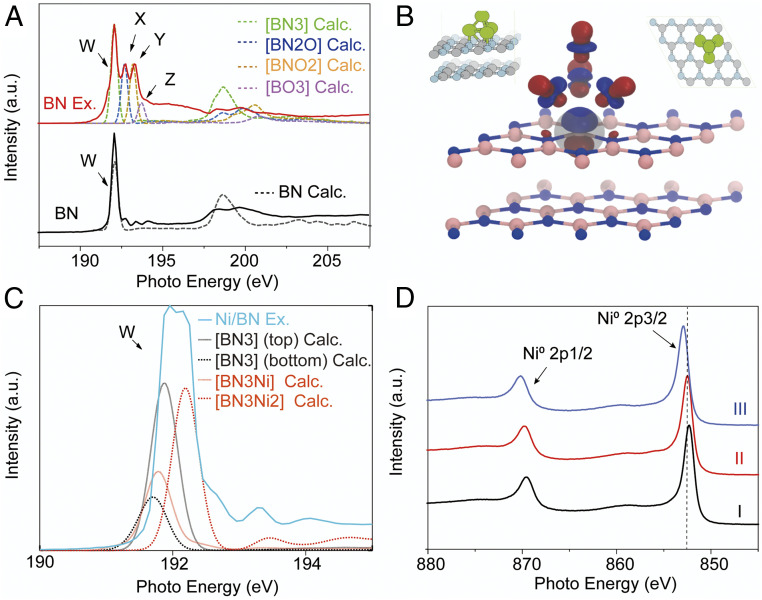
Experimental and calculated spectroscopic characteristics of pristine bulk BN, exfoliated BN nanosheets, and Ni/BN nanocomposite. (*A*) Experimental (solid lines) and calculated (dashed lines) X-ray absorption spectroscopy (XAS) spectra of pristine BN, BN nanosheets around the B K-edge. (*B*) The main contributing core-excited state to the W peak of the B atom in the BN_3_–Ni4 bonding configuration (*Bottom*). (*Inset*) Top (*Top Right*) and side (*Top Left*) views of the structure of Ni_4_ adsorbed on pristine h-BN. (*C*) Calculated B K-edge XAS spectra of B atoms in bonding configurations BN_3_, BN_3_–Ni, and BN_3_–Ni_2_ within the structure of Ni_4_ adsorbed on pristine h-BN (dotted lines), together with experimental Ni-decorated exfoliated h-BN XAS spectra (green). (*D*) XAS X-ray photoelectron spectroscopy (XPS) of nickel nanoclusters deposited on BN nanosheets with various particle sizes (I, ∼10 nm; II, ∼5 nm; III, ∼1.5 nm).

To further reveal the nature of the defects and the interaction between Ni NPs and BN nanosheets, we performed XAS on the pristine BN, exfoliated BN nanosheets, and Ni/BN nanocomposite. Fig. 4*A* shows the corresponding π-transition around the B K-edge [B(1s → π*)]. The dominant characteristic peak at 192.0 eV (labeled as W in the *Bottom* panel of Fig. 4*A*) represents a core exciton with a π-like final-state wave function. This is a specific fingerprint of sp^2^ hybridized B atoms in the hexagonal BN network, i.e., B is bonded to three N atoms in the planar configuration (59, 60). Additional peaks, labeled as X, Y, and Z (located at 192.7, 193.4, and 194.1 eV, respectively), are assigned to local defects or crystal lattice deformation around the excited B atom. A significant increase in peaks X and Y (at 192.6 and 193.3 eV) is observed after exfoliation (mid panel of Fig. 4*A*). The origin of these XAS spectra differences was investigated using spin-polarized, dispersion-corrected DFT (see computational details in *SI Appendix*). We carried out a comparative analysis of the formation energies and XAS signatures of several possible vacancies (V_X_) and terminations (see detailed results in *SI Appendix*, section S2). In h-BN surfaces, O-terminated triangular N/B_x_N_y_ vacancies V_BxNy_:O (Fig. 1 and *SI Appendix*, Fig. S11) are thermodynamically favorable to create, while generating V_B_, V_N_, hydrogen-terminated V_B_, or carbon-terminated V_N_ defects is endothermic (*SI Appendix*, Table S2), in agreement with previous theoretical predictions (61–63). The calculated B K-edge spectra of oxygen-terminated triangular defects are in excellent agreement with X, Y, and Z resonances (Fig. 4*A*, dotted line). The substitution of N by O in the bonding configuration of a B atom leads to an increase in the boron oxidation state, stabilizing the 1s core state and hence causing a proportional blue shift in the 1s-π* transition (61). Thus, peaks X, Y, and Z in the experimental XAS spectra (Fig. 4*A*) correspond to B bonded to two N and one O atom [BN_2_O], one N and two O atoms [BNO_2_], and three O atom [BO_3_] environments. When boron is bonded to less oxidizing elements than O, such as C, H, (or Ni), the 1s-π* W peak shifts to lower energies by close to 1 eV (*SI Appendix*, Figs. S12–S14). The formation of the non–O-terminated defects considered here is thermodynamically unfavorable and their spectral signature (red shifts of the main peak) was not detected experimentally (Fig. 4*A*). The predicted triangular defects are also in agreement with the TEM images of the exfoliated surface (Fig. 2*B*).

Furthermore, when Ni nanoclusters were deposited on the defective BN nanosheets, peak W was found to be much broader (Fig. 4*C*) and peak X and peak Y intensity decreases again (Fig. 4*C*). Peak fitting shows that the peak consists of two individual peaks, with the right one being shifted 0.3 eV to higher energy (*SI Appendix*, Fig. S5). The N 1s → π* region of different BN samples was also recorded (*SI Appendix*, Fig. S4). However, no clear peaks were found around the excitonic peak centered at 402.1 eV, indicating the relatively constant bonding configuration around the N atoms in the samples. For the Ni/BN system, calculated formation energies indicate that capping vacancies with a Ni cluster fails to alleviate the destabilization caused by the undercoordinated border atoms (*SI Appendix*, Table S2). When Ni sits on a pristine h-BN layer, the calculated XAS spectra (Fig. 4*C* and *SI Appendix*, Fig. S14) indicate that those B atoms in the top h-BN layer experience metallic screening that leads to a slight red-shifting of the W peak by close to 0.1 eV. On the other hand, the core-excited state shows some degree of delocalization over the Ni cluster for those B atoms that lie directly underneath it (Fig. 4*B*). This causes a decrease in the electron-hole attraction and thus in the exciton binding energy, which leads to a blue shift of the peak by close to 0.3 eV with respect to the pristine layer peaks. This may partly explain the observed broadening of peak W in the spectra of Ni/BN in Fig. 4*C*. Additionally, the smaller intensity ratio between the π* and the σ* peaks (at ∼200 eV) implies that the sample became more polycrystalline, possibly because of the formation of smaller flakes.

In addition, X-ray photoelectron spectroscopy (XPS) measurement was carried out to study the electronic properties of various-sized Ni nanoclusters on boron nitride nanosheets in the Ni 2p region (Fig. 4*D* and *SI Appendix*, Fig. S6). Both the main Ni 2p3/2 peak and Ni 2p1/2 peak, corresponding to metallic Ni species, gradually shifted to higher binding energies in a magnitude of ∼0.7 eV with Ni particle size decreasing from ∼10 nm (852.3, 869.5 eV) to ∼1.5 nm (853.0, 870.2 eV). The detailed peak fitting on the typical spectrum was presented in *SI Appendix*, Fig. S6. The observed charge deficiency on smaller Ni nanoclusters more likely results from light surface oxidation, since the formation of NiO would lead to a larger shift (close to 1.1 eV) and a change in crystal structure, contradictory with the Ni(111) facets observed in TEM images of the nanoclusters (Fig. 2*E*) (64). We carried out a series of DFT-based full core hole calculations on a lightly oxidized model nanocluster (Ni_38_O_3_) deposited on h-BN (*SI Appendix*, Fig. S16). Although the observed NPs are larger, Ni_38_ was selected for being qualitatively representative of the experimentally observed fcc 1.5-nm nanoclusters, which show (111) facets, while being computationally tractable. Oxidation and close interaction with the h-BN surface can induce shifts in the excitation energy of close to 0.5 eV, which may partly explain the shift observed for only the smallest set of nanoclusters. Nonetheless, partial surface oxidation is expected to vanish under hydrogen-rich catalytic environments.

### DFT Calculations on Ni–Support Interactions.

The nucleation and growth of Ni nanoclusters on exfoliated BN leads to BN nanoflakes that are smaller and less defective (i.e., edge-rich), according to the spectroscopic results shown above. The significant decrease in the concentration of BN O-terminated defects in the nanoflakes is roughly estimated to drop from 0.35 to 0.18 V_BN3_:O/nm^2^, compared to the initial 0.11 V_BN3_:O/nm^2^ in bulk BN (*SI Appendix*, Table S3). In order to understand the interactions between Ni nanoclusters and pristine and defective h-BN surfaces, we carried out a series of DFT calculations. Nanocluster nucleation may preferably take place at defects (*SI Appendix*, Table S4): While single Ni atoms rather adsorb on top of B on the pristine surface (*E*_ads_ = −0.47 eV), adsorption is slightly more favorable on the BN_2_O defects (*E*_ads_ = −0.50 eV). The decrease in defect concentration with Ni deposition can be explained by Ni nucleation at defects: Ni affinity for oxygen possibly leads to the formation of B_2_O_3_/B(OH)_3_ species that would be washed away during sample preparation (before the XAS characterization), and a new pristine or H-terminated BN edge. Such edges form step-like structures in the BN nanoflakes, hereafter referred to as “pits.” However, the nanocluster adsorption site preference is size dependent. Larger Ni clusters (Ni_38_) have a stronger interaction with the pristine surface than with the defected surface (0.44 V_BN3_:O/nm^2^), with adsorption energies of −4.25 and −3.53 eV, respectively, which further increase to −4.98 eV when nearby N-H–terminated edges are present (65), as in the pit structures (see Fig. 1 and *SI Appendix*, Fig. S17). Based on these results, we propose the “pit” model structure for the Ni/BN system: Ni nanoclusters occupy pristine regions of the BN nanoflakes and interact with nearby BN edges. Other configurations such as those involving neighboring nanoflakes or nanoclusters that are larger than the pits are also compatible with our experimental results. Hence, NPs grown on exfoliated and defective BN are likely considerably more immobilized than those grown on bulk h-BN, lowering the probability of sintering and thus allowing to maintain NP size. Nanocluster size is key for catalysis (see below). It should be noted that, although the nanoclusters are strongly chemisorbed on BN, charge transfer is minimal (below 0.2 e, according to Bader analysis) and the electronic structure of the nanoclusters remains largely unmodified (*SI Appendix*, Fig. S15), in agreement with the literature (66–68).

### Catalytic Performance for Methanol Dehydrogenation.

**Fig. 5.**
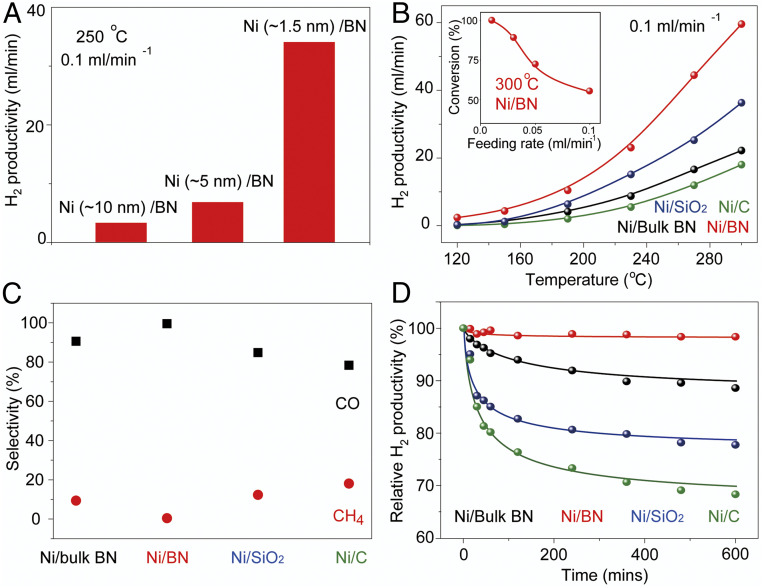
Catalytic performance of Ni nanoclusters on various substrates for methanol dehydrogenation. (*A*) Hydrogen productivity of Ni/BN nanocomposite with various Ni nanocluster sizes. (*B*) Temperature-dependent hydrogen productivity of Ni nanoclusters on various substrates at the methanol feeding rate of 0.1 mL/min. (*C*) Selectivity and (*D*) the long-term durability of various catalysts for methanol dehydrogenation (methanol feeding rate, 0.1 mL/min; N_2_ feeding rate, 30 mL/min; temperature, 300 °C).

To evaluate the catalytic performance of different types of Ni nanocluster-based catalysts for methanol dehydrogenation from methanol, we performed high flow rate catalytic experiments under atmospheric pressure in a fixed-bed continuous flow reactor. In a typical catalytic measurement, the catalysts samples were packed into reactor tubes and treated by nitrogen (20 mL⋅min^−1^) at 150 °C for 20 min to remove unexpected impurities. Then liquid methanol was pumped into the heating chamber with a feed rate of 0.1 mL/min. In the heating chamber, methanol vapor was mixed with carrier gas N_2_ before feeding into the reactor tube. The final products were analyzed by a gas chromatography instrument equipped with a thermal conductivity detector (TCD) and a flame ionization detector (FID). Fig. 5*A* shows the size effect of Ni nanoclusters toward hydrogen productivities of Ni/BN catalysts under a high liquid methanol feeding rate (0.1 mL/min). This result shows that hydrogen productivity significantly decreased when increasing the nanocluster size from ∼1.5 to ∼10 nm (*SI Appendix*, Fig. S7) at the same temperature of 250 °C, which demonstrates that the smaller size of Ni nanoclusters favors the dehydrogenation of methanol. The higher surface area of smaller nanoclusters (under the same Ni loading amount) implies that more Ni active sites are available for the dehydrogenation reaction, increasing hydrogen productivity. In addition, smaller nanoclusters show better selectivity during the reaction (*SI Appendix*, Fig. S8). Methanol decomposed mainly to hydrogen and carbon monoxide over the nickel catalysts. Methane was reported as the by-product.

We further investigated the support effect of the Ni/BN catalyst for methanol dehydrogenation. The temperature dependence of methanol decomposition over various Ni catalysts directly quantifies the support effect on the hydrogen productivity in Fig. 5*B*. Compared to those supported on activated carbon (∼18 mL/min), bulk h-BN (∼24 mL/min), and SiO_2_ (∼32 mL/min), the catalyst of Ni nanoclusters deposited on defective BN nanosheets displays the highest hydrogen productivity (∼60 mL/min at 300 °C at the feeding rate of 0.1 mL/min), which shows that BN nanosheets are excellent support for Ni nanoclusters. In addition, as shown in Fig. 5*B*, the Ni/BN catalyst sample shows decent activity for the methanol decomposition when the reaction temperature is as low as 120 °C, which further confirms the support effect of exfoliated BN nanosheets has a great influence on controlling the formation of smaller-sized nanoclusters, which generates higher catalytic activity. The results of control experiments show that both the BN nanosheets and bulk h-BN supports did not exhibit detectable conversions of methanol. We also measured the methanol dehydrogenation performance of the Ni/BN catalyst at different methanol feeding rates (*Inset* of Fig. 5*B*). The methanol conversion rate is higher at a lower feeding rate and, for example, could reach 100% (totally converted) when the feeding rate is 0.01 mL/min. The catalytic activity of Ni/BN for methanol dehydrogenation was further compared with some other transition metal-based catalysts reported previously on TOF calculated from hydrogen productivity, where the performance of Ni/BN is among the best (*SI Appendix*, Table S1). Another merit for the Ni/BN catalyst is its high catalytic selectivity. As shown in Fig. 5*C*, the selectivity to H_2_ and CO is nearly 100% for Ni/BN catalyst. However, methane was detected when Ni nanoclusters were deposited on bulk h-BN, SiO_2_, and activated carbon. The deposition of Ni nanoclusters on defective BN nanosheets also significantly improves the catalytic stability of the dehydrogenation. Fig. 5*D* shows the long-term durability of various catalysts for methanol dehydrogenation. No obvious deactivation was found on Ni/BN catalyst after testing for over 10 h, indicating the feasibility and robustness of Ni/BN catalyst for methanol dehydrogenation. The TEM images of the used catalyst in *SI Appendix*, Fig. S9 further confirms the structural stability after testing. As a comparison, only 88.8%, 78.0%, and 68.7% of activity was maintained when Ni nanoclusters were deposited on bulk h-BN, SiO_2_, and activated carbon, respectively. Moreover, the Ni/BN catalyst shows the universality of efficiently decomposing some other kinds of alcohol, especially ethanol, to form the products of hydrogen and aldehydes with high productivity (*SI Appendix*, Fig. S10).

**Fig. 6.**
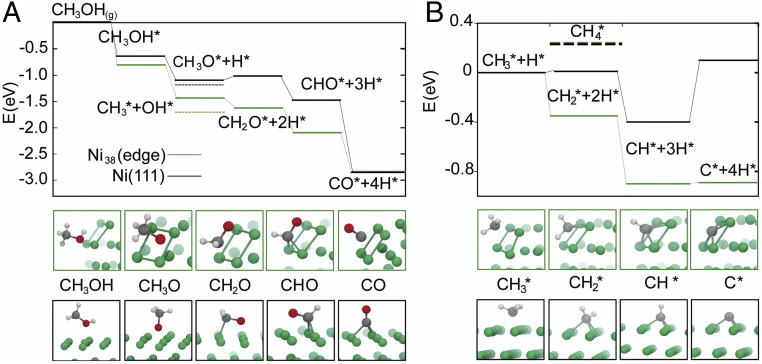
(*A*) Comparison of the relative energies of the methanol dehydrogenation and CHx. (*B*) Intermediates in the edges (green) of the Ni38 NP, and on Ni(111) (black).

Finer detail on the size dependence of the activity and selectivity for methane can be obtained with dispersion-corrected DFT calculations (see *SI Appendix*, section S1 for details). We used a Ni38 cluster and a Ni(111) extended flat surface to model a small and a large nanoparticle, respectively. First, we compared the enthalpic drive of the main methanol dehydrogenation pathway on Ni38 and Ni(111) in order to understand how the specific sites in NP edges and terraces affect the energetics of the reaction. The results are summarized in Fig. 6*A*. In most Ni surfaces (69, 70), methanol dehydrogenation begins with methanol adsorption and O–H bond cleavage leading to surface methoxy, CH_3_O*, where * indicates the species is adsorbed on the surface. Further dehydrogenation produces formaldehyde (CH_2_O*), formyl (HCO*), and finally CO and two molecules of H_2_ (69, 70). The highest H_2_ production on smaller NPs can be traced to two key differences. First, the affinity for methanol of the sites at the Ni(100)/Ni(111) edge on Ni38 is higher than that of Ni(111), with *E*_ads_ of −0.81 and −0.65 eV, respectively. Second, the dehydrogenation pathway is fully downhill on NP edges. On Ni(111), CH_3_O dehydrogenation is endothermic and the rate-determining step (70) of this pathway. However, formaldehyde chemisorption at the Ni_38_ edge sites, driven by their fourfold symmetry and higher d-electron density of the Ni undercoordinated atoms, boosts the adsorption energy from −0.99 eV on Ni(111) to −1.66 eV (*SI Appendix*, Tables S5 and S6), rendering the CH_3_O dehydrogenation step exothermic.

**Fig. 7.**
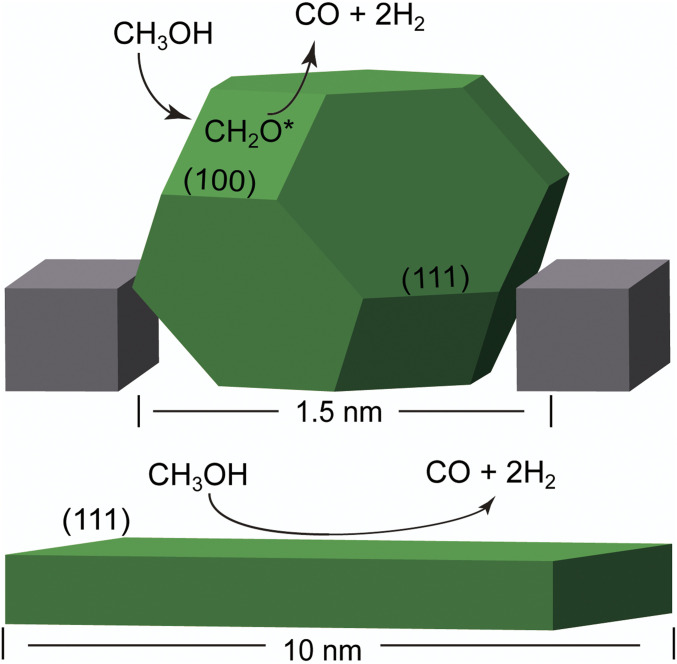
Scheme summarizing nanoparticle size effects on methanol dehydrogenation: dehydrogenation with enhanced formaldehyde adsorption on model Ni NP on BN pit (*Top*) and on Ni(111) (*Bottom*).

The observed selectivity may result directly from the fully exothermic nature of the dehydrogenation pathway at the NP edges. Nevertheless, we have extended the above comparison to understand NP size effects on possible methane formation intermediates. Despite the extensive research on methanol dehydrogenation pathways, to our knowledge, there are no reports on CO/methane selectivity on methanol dehydrogenation on Ni. Studies on the selectivity for the reverse reaction (CO methanation/methanolation on the Ni(111) surface) show that favorable pathways for methane formation evolve from C–O bond dissociation in CHO and CH_2_O species, leading to CHx species and eventually methane (53). We find that C–O bond dissociation is most favorable from methanol itself (*SI Appendix*, Table S7); and that edge sites are more active in dissociating C–O bonds than Ni(111) sites. For instance, once methanol is adsorbed, C–O scission is more favorable on the NP than on the Ni(111) by 0.42 eV, in agreement with reports on lower-index Ni surfaces (71). From the CH_3_ species, the formation of methane is similarly endothermic in both structures, by 0.24 and 0.22 eV for the NP and Ni(111), respectively (Fig. 6*A*). Another pathway leading to methane formation involves CO methanation. The first step is reported to be the formation of HCO, which here is endothermic by 0.8 and 1.2 eV, respectively, for the NP and the Ni(111) surface (72, 73). Full CO methanation is quite endothermic as well at the Ni(111) surface, requiring with 2.05 eV. At the NP edge, direct CO scission is slightly endothermic (0.08 eV), although the required activation energy has been reported to be as high as 1.95 eV on the Ni(100) surface, which has sites with fourfold symmetry too (71). These results indicate that the formation of CH_4_ is more favorable on the smaller nanoclusters (*SI Appendix*, Fig. S18), in contradiction of the size-dependent selectivity observed in experiment. Nonetheless, there is a key difference between the NP edge and the Ni(111) surface. The formation of C* species from the dehydrogenation of CHx species and CO dissociation is much more favorable at the fourfold sites (Cf) and subsurface (Csub) of the Ni NP than at the Ni(111) surface (Fig. 6*B*). For Ni_38_ edge sites, CH_3_ dehydrogenation to C* is fully downhill and exothermic by 0.89 eV, while methane formation is endothermic by 0.24 eV. Both reactions are lightly endothermic at the Ni(111) surface, by 0.10 and 0.22 eV. Fourfold sites have been reported to greatly facilitate C* diffusion to the subsurface at Pd NP edges (74). According to Aleksandrov et al. (75), subsurface carbon tends to form Cn chains (hence, coking) at the Ni(111) surface. On the other hand, smaller NPs are able to sustain a larger amount of Csub due to their structural flexibility (75). Additionally, the CO adsorption energy on Ni38/Csub and Ni38/Cf decreases by more than 0.4 eV, with *E*_ads_ of −1.63 and −1.48 eV, respectively (*SI Appendix*, Fig. S19). A similar effect has been reported on Pd(111) surfaces (76). Consequently, the tendency of CO to remain on the surface of small NPs, blocking adsorption sites and/or reacting to form methane, can be significantly reduced in the presence of subsurface carbon, modifying the thermodynamic sink due to increased CO release. These calculations indicate that, besides the possible (but needing further exploration) role of subsurface C, increased formaldehyde adsorption and favorable CH_X_ dehydrogenation can be the origin of the selectivity of the 1.5-nm NPs, compared to large NPs. Key to this phenomenon is the availability of undercoordinated fourfold sites. A scheme summarizing these results is shown in Fig. 7. The experimental results are consistent with the computational analysis that the metal NP size plays a vital role during the catalytic reaction. Therefore the ∼1.5-nm Ni NP-based catalysts were chosen as the model for methanol dehydrogenation in this study. We further investigated the support effect of the Ni/BN catalyst for methanol dehydrogenation. The temperature dependence of methanol decomposition over various Ni catalysts directly quantifies the support effect on the hydrogen productivity in Fig. 5*B*. Compared to those supported on activated carbon (∼18 mL/min), bulk h-BN (∼24 mL/min), and SiO_2_ (∼32 mL/min), the catalyst of Ni nanoclusters deposited on defective BN nanosheets displays the highest hydrogen productivity (∼60 mL/min at 300 °C at the feeding rate of 0.1 mL/min), which shows that BN nanosheets are excellent support for Ni nanoclusters. In addition, as shown in Fig. 5*B*, the Ni/BN catalyst sample shows decent activity for the methanol decomposition when the reaction temperature is as low as 120 °C, which further confirms that the support effect of exfoliated BN nanosheets has a great influence on controlling the formation of smaller-sized nanoclusters, which generates higher catalytic activity. The results of control experiments show that both the BN nanosheets and bulk h-BN supports did not exhibit detectable conversions of methanol. We also measured the methanol dehydrogenation performance of the Ni/BN catalyst at different methanol feeding rates (*Inset* of Fig. 5*B*). The methanol conversion rate is higher at a lower feeding rate and, for example, could reach 100% (totally converted) when the feeding rate is 0.01 mL/min. The catalytic activity of Ni/BN for methanol dehydrogenation was further compared with some other transition-metal based catalysts reported previously on TOF calculated from hydrogen productivity, where the performance of Ni/BN is among the best (*SI Appendix*, Table S1). Another merit for the Ni/BN catalyst is its high catalytic selectivity. As shown in Fig. 5*C*, the selectivity to H_2_ and CO is nearly 100% for Ni/BN catalyst. However, methane was detected when Ni nanoclusters were deposited on bulk h-BN, SiO_2_, and activated carbon. The deposition of Ni nanoclusters on defective BN nanosheets also significantly improves the catalytic stability of the dehydrogenation. Fig. 5*D* shows the long-term durability of various catalysts for methanol dehydrogenation. No obvious deactivation was found on Ni/BN catalyst after testing for over 10 h, indicating the feasibility and robustness of Ni/BN catalyst for methanol dehydrogenation. The TEM images of the used catalyst in *SI Appendix*, Fig. S9 further confirms the structural stability after testing. As a comparison, only 88.8%, 78.0%, and 68.7% of activity were maintained when Ni nanoclusters were deposited on bulk h-BN, SiO_2_, and activated carbon, respectively. Moreover, the Ni/BN catalyst shows the universality of efficiently decomposing some other kinds of alcohol, especially ethanol to form the products of hydrogen and aldehydes with high productivity (*SI Appendix*, Fig. S10).

The highest productivity, selectivity, and durability of Ni/BN with respect to Ni/bulk BN catalyst samples indicate the catalytic enhancement likely arises due to the stronger interfacial interactions in the exfoliated BN pits (see above), which prevent nanoparticle growth through sintering. As shown above, DFT calculations reveal that smaller nanoparticles adsorb methanol and methanol dehydrogenation intermediates more strongly, leading to higher productivity, and can sustain Csub. Although these results indicate that Csub weakens CO adsorption, and hence could influence selectivity and reduce CO poisoning, future experimental and computational work on the reaction network is necessary to confirm the presence of subsurface carbon and elucidate in detail its role in the mechanism(s) with increasing NP size. Other explanations for the selectivity such as H spillover to the BN surface were deemed unlikely based on the adsorption of H on BN (either pristine or defected) being thermodynamically unfavorable with *E*_ads_ of 2.12 and 2.24 eV, respectively. Strong electronic metal–support interactions due to vacancies (24, 25) in the defected BN at the catalytic temperatures used here seem improbable too, as per the thermodynamic and spectroscopic characterization described earlier in the manuscript. Alternatively, a recent study on a similar system (albeit with larger Ni NPs) emphasizes the role of the B-O and B(OH) moieties at BN edges as possible CO spillover sites, although this is not contemplated in our hypothesis (54).

## Conclusion

In summary, we reported the design and synthesis of ultrasmall nickel nanoclusters (∼1.5 nm) deposited on oxygen vacancy-abundant BN nanosheet (Ni/BN) catalysts with excellent methanol dehydrogenation activity and selectivity. The surface modification of the BN substrate has been demonstrated to be a very effective knob to tune the metal–support interaction both in the nucleation and growth of ultrasmall Ni nanoclusters, and further facilitates catalytic action and heightens overall performance by means of interacting with the size sustained metal nanoclusters. The catalytic results demonstrated that the size effect of Ni nanoclusters and interfacial engineering of the support play important roles in both the activity and the selectivity of the catalyst. We performed DFT calculations to reveal the origin of the high productivity, high selectivity, and high durability exhibited with the Ni/BN nanocatalyst and elucidate its correlation with nanocluster size, and support–nanocluster interactions. The size dependence of the productivity and selectivity was traced to the higher adsorption of methanol and formaldehyde on the NP edges. The superior catalytic performance of productivity, selectivity, stability, and universality shown with the Ni/BN catalyst proves its high potential for industrial application. More importantly, this in-depth study attracts more attention to the synergistic effect of metal–support interaction both during the catalysts’ synthesis and functional process, which paves the way and possesses the great referential significance for further rational design of highly efficient catalysts for catalysis application.

## Materials and Methods

### Exfoliation of BN Sample.

h-BN powder (0.5 g) in quartz beaker was ramped to 800 °C in a furnace under air. After calcinated for 5 min at 800 °C, the sample was immediately immersed into a liquid nitrogen-filled Dewar bottle. The liquid nitrogen would be gasified completely due to the large amplitude of temperature fluctuations. The sample was then recollected and the steps were redone as described above. The total repeating times were 10. After that, the as-obtained BN powder was added to THF, sonicated for 40 min, and then centrifuged at 1,000 rpm for 10 min to precipitate excess large particles. The supernatant was dried in a vacuum oven and then transferred to argon glovebox. For the lithium intercalation, 0.18-g dried BN nanosheets were dispersed in THF dissolved with lithium (0.036 g)–naphthalenide (0.64 g) solution. The solution was heated at 65 °C with stirring for another 5 h and then sonicated for 40 min.

### Deposition of ∼1.5-nm Ni Nanoclusters on Various Nanosheets.

Bis(cyclopentadienyl)nickel(II) (0.064 g) was dissolved in THF (10 mL) at room temperature with stirring. The solution was then quickly injected into the BN-lithium naphthalenide solution and stirred for 30 min. The resultant solution was precipitated by adding acetonitrile and further centrifugation. The precipitation was washed by THF three times to remove the by-products and then dried under vacuum overnight for further use. The method to deposit ∼1.5-nm Ni nanoclusters on the other kinds of substrate was similar as described above but using various substrate materials.

### Deposition of ∼5-nm Ni Nanoclusters on BN Nanosheets.

The BN nanosheets obtained by step 1 were separated via centrifugation, washed with ACN three times, and dried in vacuum. Then 0.18-g BN nanosheets and 0.064 g of Bis(cyclopentadienyl)nickel(II) were dispersed/dissolved in THF (10 mL) at room temperature with stirring. Afterward, 0.026 g of sodium borohydride dissolved in 5 mL of ACN was then quickly injected into the BN-(Cp)_2_ Ni solution and stirred for 30 min at room temperature. The resultant solution was precipitated by centrifugation, washed by ACN three times to remove the by-products, and then dried under vacuum overnight for further use.

### Deposition of ∼10-nm Ni Nanoclusters on BN Nanosheets.

The BN nanosheets obtained by step 1 were separated via centrifugation, washed with ACN three times, and dried in vacuum. Then 0.18-g BN nanosheets and 0.064 g of Bis(cyclopentadienyl)nickel(II) were dispersed/dissolved in THF (10 mL) at room temperature with stirring. Afterward, 0.026 g of sodium borohydride in 5 mL of ACN was then slowly dropped into the BN-(Cp)_2_ Ni solution with stirring at 65 °C in 10 min and then kept for another 30 min. The resultant solution was precipitated by centrifugation, washed by ACN three times to remove the by-products, and then dried under vacuum overnight for further use.

### Catalytic Measurements.

In a typical catalytic measurement, 200-mg catalyst samples were packed into reactor tubes and treated by nitrogen (20 mL⋅min^−1^) at 150 °C for 20 min to remove unexpected impurities. Then liquid methanol was pumped into the heating chamber with various feed rates. In the heating chamber, methanol vapor was mixed together with carrier gas N_2_ (20 mL/min) before feeding into the reactor tube. The products were analyzed online by HP 5890 GC (hayesep D column and hayesep Q column) equipped with TCD and FID detector.

### Materials Characterizations.

The general TEM images were obtained by transmission electron microscope (JEOL 2100F) with an accelerating voltage of 200 kV. The high-resolution spherical aberration-corrected TEM images were obtained by ThemIS microscope at 60 kV. FTIR spectra were obtained with an Agilent Cary 630 spectrometer. Raman spectra were obtained by LabRAM. PXRD patterns were acquired with a Bruker AXS D8 Discover GADDS X-ray diffractometer, using Cu and Co Kα radiation. XAS measurements at boron and nitrogen K-edges were carried out at beamlines 7.3.1 and 8.0.1.1 at the Advanced Light Source, Lawrence Berkeley National Laboratory. XPS was measured by K-Alpha Plus XPS/UPS. The actual amounts of nickel were confirmed by elemental analysis using inductively coupled plasma optical emission spectroscopy (Varian ICP-OES 720 Series).

### Computational Methods.

All model structures were optimized using periodic, spin-polarized, dispersion-corrected DFT as implemented in Quantum ESPRESSO (77). The PBE (78) exchange-correlation functional was used together with the D3 (79) dispersion correction and ultrasoft pseudopotentials to model the core electrons. Converged values of the density and wavefunction cutoff (43.0 and 384.0 Ry, respectively) were used. Unless otherwise mentioned, a gamma-point *k*-point grid was found to provide converged results due to the relatively large size of the supercells. A Gaussian smearing on 0.0019 Ry was used to improve the convergence of the self-consistent electronic energy.

The atomic positions and lattice vectors of bulk boron nitride were relaxed using a 6 × 6 × 6 *k*-point grid. Then, two h-BN structures were generated: a single-layer, 4 × 4 supercell (*a* = 10.03 AA), to investigate defect formation; and a bilayer 6 × 6 supercell (*a* = 15.05 AA), to investigate support–nanocluster interactions.

In order to compute defect formation energies, structures with target defects were created from the 4 × 4 h-BN monolayer structure. All structures were relaxed until the total energy changed less than 1.0^−4^ Ry and the forces were below 1.0^−3^ a.u. Defect formation energies *E*_f_ were calculated as per ref. 80. Since all systems here considered were charge-neutral:

$$E_{\text{f}}\left[\text{X}\right]=E\left[\text{X}\right]–E\left[\text{host}\right]–\Sigma \left(n_{i}\mu_{i}\right),$$

where *E*[X] and *E*[host] are the total energies of the defective and pristine system, *n*_*i*_ is the net amount of particles of type *i* added (*n*_*i*_ > 0) or removed (*n*_*i*_ < 0), which are assumed to be exchanged with a reservoir with chemical potential *μ*_*i*_. The reference phases of N, O, H, C, and B are taken to be N_2(g)_, O_2__(g)_, H_2__(g)_, CH_4__(g)_, and orthorhombic boron (*a* = 4.88, *c* = 12.49), respectively.

Many-body XAS spectra of the optimized structures of interest were calculated using the excited core-hole approach as implemented in MBXAS (81). The individual spectra of target B or N atoms was calculated using a finer 5 × 5 × 5 *k*-point grid and the same wavefunction, and density cutoffs, and pseudopotentials (with exception of the excited-atom pseudopotential) as were used in the structure optimization. A peak broadening of 0.2 eV was used. Support–nanocluster interactions were investigated using Ni_38_ as a model NP and the previously optimized bilayer, 6 × 6 boron nitride supercell. Ni_38_ was selected for being qualitatively representative of the experimentally observed 1.5-nm nanoclusters, which show (111) facets, while being computationally affordable. The structure was generated based on Sutton–Chen global minima search (82). Two layers of h-BN were deemed sufficient to model the system due to the weak interactions between the layers (67).

At last, adsorption energies of methanol decomposition and CO methanation intermediates were calculated as follows:

$$E_{\text{ads}}=E{\left[\text{A}*\right]}_{m}-\left(E\left[\text{A}\right]+E\left[*\right]\right),$$

where *E*[A*]_*m*_ is the total energy of species A adsorbed on the Ni_38_ surface at site *m*, *E*[A] is the total energy of A in the gas phase, and *E*[*] is the total energy of bare Ni_38_ nanocluster. All adsorbates were considered as isolated species, that is, we assume a low coverage situation. The h-BN support was omitted in these calculations, since the change in adsorption energy observed in methanol and CO was below 0.01 and 0.07 eV, respectively. For the Ni(111) surface, the lattice parameters and atomic positions of the bulk unit cell were optimized first. All supercells were made large enough in the direction perpendicular to the surface (20 to 27 AA) so as to avoid spurious interactions between periodic images.

## Supplementary Material

Supplementary File

## Data Availability

All study data are included in the article and *SI Appendix*.
