# Supplementary material for: Enhanced and stabilized hydrogen production from methanol by ultrasmall Ni nanoclusters immobilized on defect-rich h-BN nanosheets
Source: Proc Natl Acad Sci U S A. 2020 Nov 9;117(47):29442–52. doi: 10.1073/pnas.2015897117 (PMC7703534; doi:10.1073/pnas.2015897117)
Supplement: Supplementary File [file pnas.2015897117.sapp.pdf]

PNAS
www.pnas.org

Supplementary Information for

Enhanced and stabilized hydrogen production from methanol by ultrasmall Ni Nanoclusters immobilized on defect-rich h-BN nanosheets

Zhuolei Zhang,[a†] Ji Su,[b, c†] Ana Sanz Matias,[a†] Madeleine Gordon,[a,d] Yi-sheng Liu,[e] Jinghua Guo,[e] Chengyu Song,[f] Chaochao Dun,[a] David Prendergast,[a*] Gabor A. Somorjai,[b, c *] Jeffrey J. Urban[a *]

a. The Molecular Foundry, Lawrence Berkeley National Laboratory Berkeley, CA 94720, USA

b. Materials Sciences Division, Lawrence Berkeley National Laboratory, Berkeley, CA 94720, USA

c. Department of Chemistry, University of California-Berkeley, Berkeley, California 94720, United States.

d. Applied Science and Technology Graduate Group, University of California-Berkeley, Berkeley, CA 94720, USA

e. Advanced Light Source, Lawrence Berkeley National Laboratory, Berkeley, CA 94720, USA

f. The National Center for Electron Microscopy, Lawrence Berkeley National Laboratory, Berkeley, CA 94720, USA

† These authors contributed equally to this paper.

Email: dgprendergast@lbl.gov, gasomorjai@lbl.gov, jjurban@lbl.gov

This PDF file includes:

Supplementary text

Figures S1 to S19

Tables S1 to S7

SI References

Section 1. Experimental characterization of the defective system

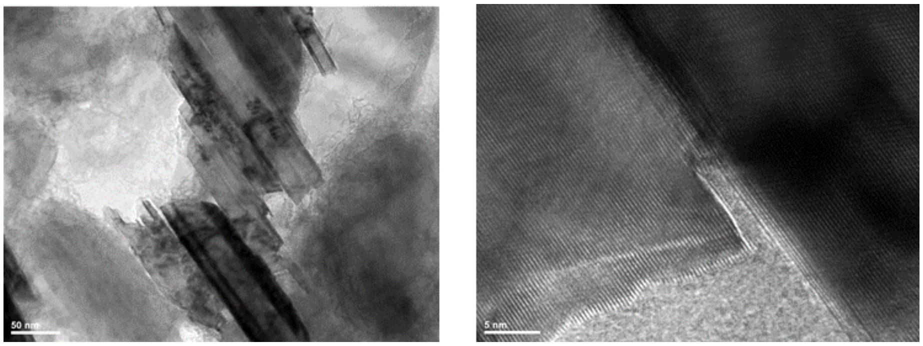

Fig. S1. TEM images of bulk BN powder.

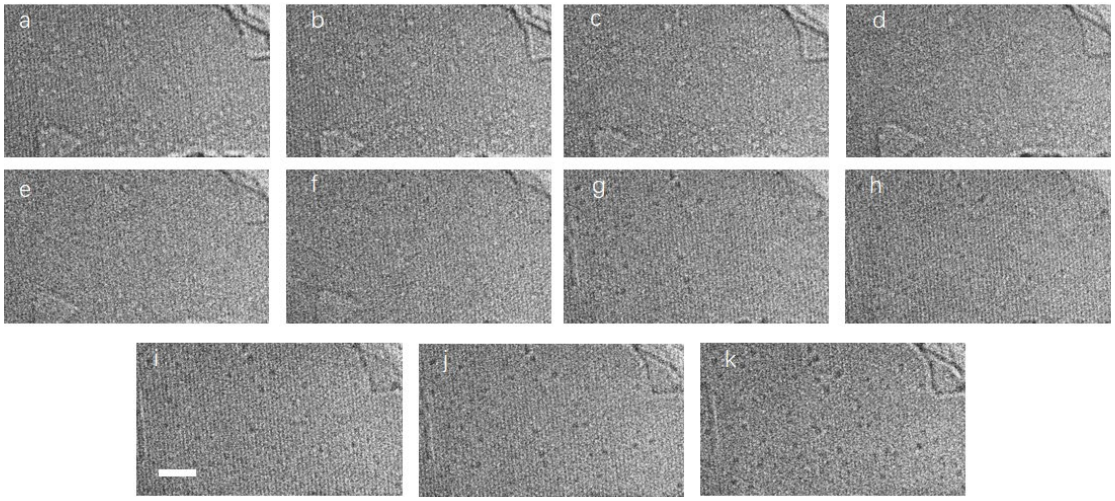

Fig. S2. High-resolution spherical aberration-corrected TEM images of exfoliated BN nanosheet at the same location but at different focus recorded through focus series with focal step of 2 nm. The gradual contrast change of the dots from white to black indicates the missing of atoms. The triangle region also indicates BN defects.

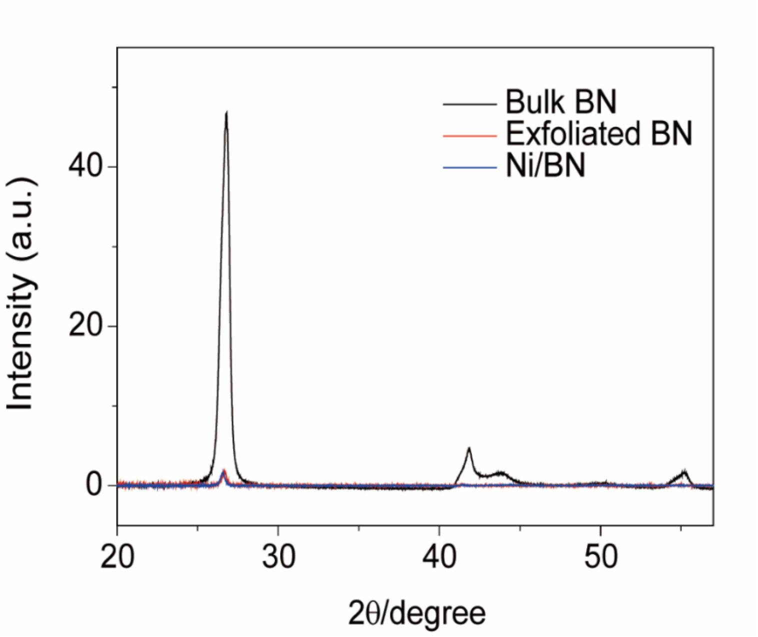

Fig. S3. X-ray powder diffraction (XRD) patterns without normalization clearly show the intensity difference between different samples.

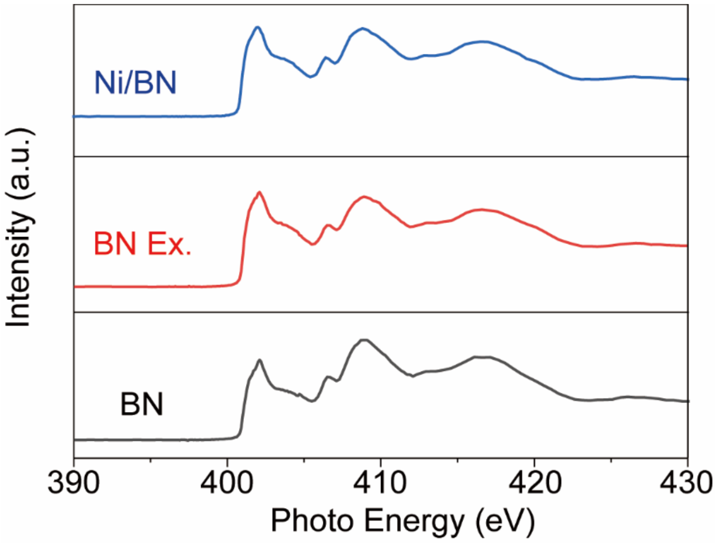

Fig. S4. X-ray absorption spectroscopy (XAS) spectra of pristine BN, BN nanosheets and Ni/BN nanocomposite around the N K-edge.

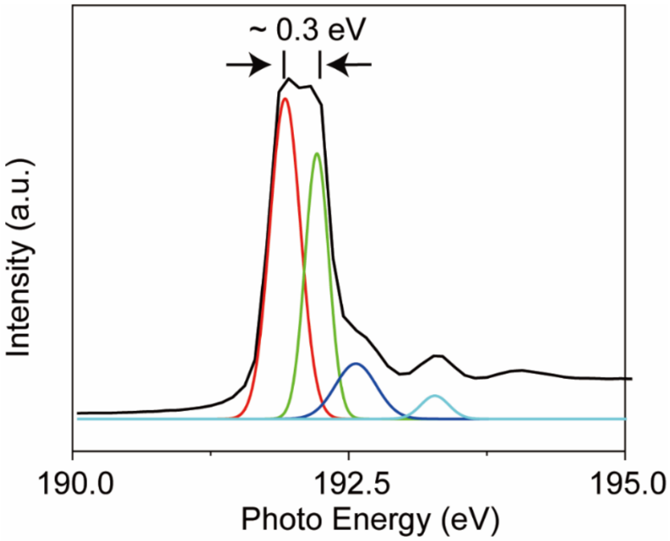

Fig. S5. Peaking fitting on the XAS spectrum of Ni/BN nanocomposite, manifesting that the W peak was consisted of two individual peaks, with the right one being shifted 0.3 eV to higher energy.

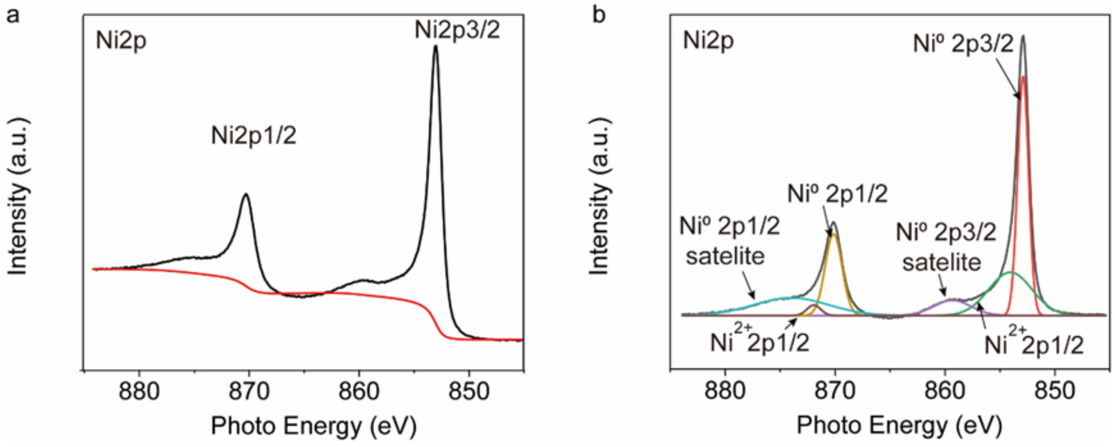

Fig. S6. (a) XPS spectrum of ~1.5 nm Nickel nanoclusters deposited on BN nanosheets (black) and baseline created by using Shirley method of XPS mode (red). (b) Detail peaks fittings and analysis on the baseline subtracted XPS spectrum of ~1.5 nm Nickel nanoclusters deposited on BN nanosheets.

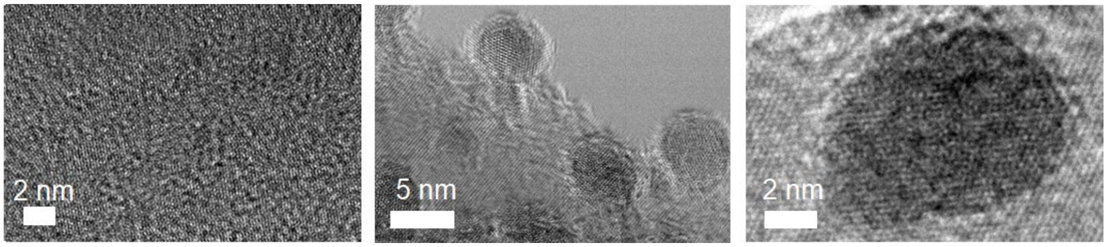

Fig. S7. (left) HRTEM image of typical ~1.5 nm Ni nanoclusters on bulk BN. (middle) HRTEM image of typical ~5 nm Ni nanoclusters on BN nanosheets (right) HRTEM image of typical ~10 nm Ni nanoclusters on BN nanosheets.

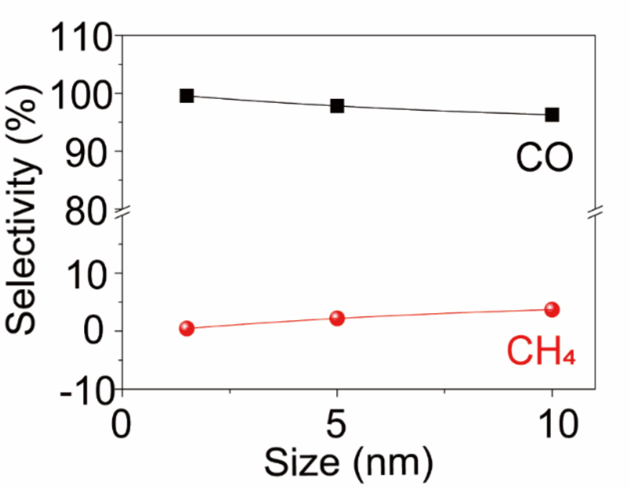

Fig. S8. Selectivity of various sized Ni/BN catalysts for methanol dehydrogenation.

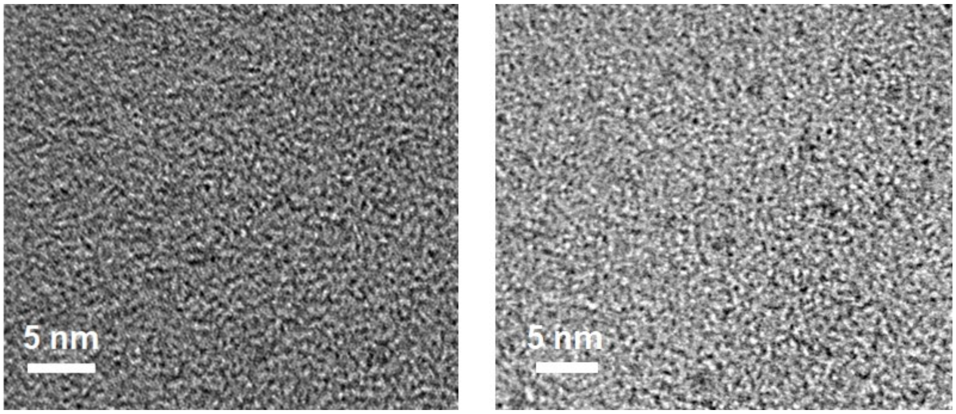

Fig.S9. TEM images of the pristine and used catalyst, which confirms the structural stability after testing.

Table S1. Catalytic performance of various catalysts for methanol dehydrogenation at 300 °C. Weight hourly space velocity (WHSV) is defined as the weight of feed flowing per unit weight of the catalyst per hour (($g\ CH_3OH\ g^{-1}$ catalysts h^{-1}). Turnover frequency (TOF) is defined as the average number of chemical reactions happened on active sites per hour. (mol CH_3OH mol^{-1}metal h^{-1})

Catalysts	WHSV	TOF	Reference
Ni/BN	23.8	237.6	This Work
$Pt/CeO_2/Al_2O_3$	15.7	946.1	R S1
Pd/CeO_2	1.0	123.4 (270 °C)	R S2
Rh/CeO_2	1.0	95.3	R S2
Ni/UDD	1.3	31.9	R S3
Commercial Cu/Cr	2.1	9.5	R S4
Co/CeO_2	1.5	13.8	R S5
$Ni/N_{0.09}CF$	15	73.4	R S6
$Ni/N_{0.09}CF$	45	131.1	R S6

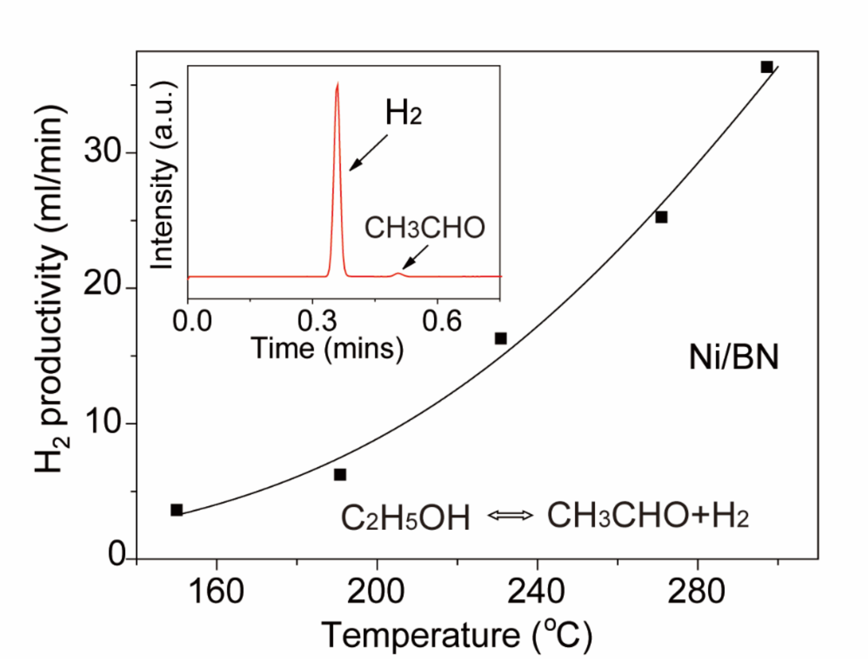

Fig. S10. The decomposition of ethanol by Ni/BN to the products of hydrogen and aldehydes with high productivity. Inset: Gas detection during the reaction by gas chromatography instrument.

Section 2. Computational characterization of the defective system

The formation energies of the defects considered here are shown in Table S2. The corresponding defect structures, together with their calculated XAS spectra are shown in Fig. S11-S15.

It should be noted that the creation of oxygen-terminated defects under Ni clusters is less favorable than in the pristine hBN surface. This effect is also somewhat size-dependent: due to the smaller flexibility of the Ni_{38} cluster, the creation of triangular defects under it is even less favorable than for Ni_4. The surface concentration of vacancies can be estimated according to a Boltzmann distribution as in eq. 4 in Ref. S7. At 300 ^oC, the maximum temperature reached in the catalytic experiments presented here, only those defects with (free) formation energies less positive than 1.1 eV can be present in non-negligible concentrations. Since the values in Table S2 are enthalpies, a qualitative assessment of the approximate weight of the entropic term of the free energy of formation of the defects should be considered. Those reactions in which net gas molecules are formed correspond to the creation of unterminated BN_3 (3/2 N_2) and N (½ N_2) vacancies. We assume here that the contribution to the entropic change from the solid phases is negligible. Given that the tabulated standard entropy of a N_2 molecule is 191.6 J/mol K (1.986 meV/mol K), the entropic contribution to the free energies of formation of the BN_3 (3/2 N_2) and N (½ N_2) vacancies at 300C are 1.71 and 0.56 eV, respectively. These are not large enough to render the any of the enthalpically unfavorable BN_3 or N vacancy formation reactions endothermic. Hence, it seems unlikely that there will be unterminated vacancies at the boron nitride surface at the temperatures used in the catalytic environment.

Table S2. Formation energies, E^f, (in eV, see computational methods) of the series of considered B, N and BN_3 vacancies at the hBN surface terminated with O, H, C and/or Ni atoms. The host states for the top part and bottom parts of the table are, respectively, the pristine hBN double layer, and the pristine hBN double layer with the Ni_X (x=4, 38) adsorbed on top. The last columns indicates those structures for which the XAS spectra was calculated.

Vacancy	Termination	E^f (per vacancy)	E^f (per edge atom)	XAS
N	-	7.63	2.54	
BN_3	-	15.54	2.59	
BN_3	O (BN_2O)	−5.43	−1.81	*
BN_3	O (BNO_2)	−5.53	−1.84	*
BN_3	O (BO_3)	−5.61	−1.87	*
N	OH (BN_2OH)	−1.13	−1.13	*
BN_3	O_2H_2 (BN_2O, BN_2H)	−0.41	−0.10	*
N	C (BN_2C)	5.58	5.58	
N	CH (BN_2CH)	3.91	3.91	*
B	H_3 (BN_2H)	2.35	0.78	
BN_3	Ni_4 (BN_2--Ni, BN_3--Ni)	8.98		*
N	Ni_4 (BN_2--Ni, BN_3--Ni)	3.43		*
N	Ni_4H_3 (BN_2H, BN_2H--Ni)	3.68		
B	Ni_4 (BN_3--Ni)	4.79		*
B	Ni_4H_3 (BN_3--Ni)	2.44		*
N	Ni_{38} (BN_2--Ni, BN_3--Ni)	3.63		
BN_3	O , Ni_{38} (BN_2O, BN_2O--Ni)	−4.69	−1.56	

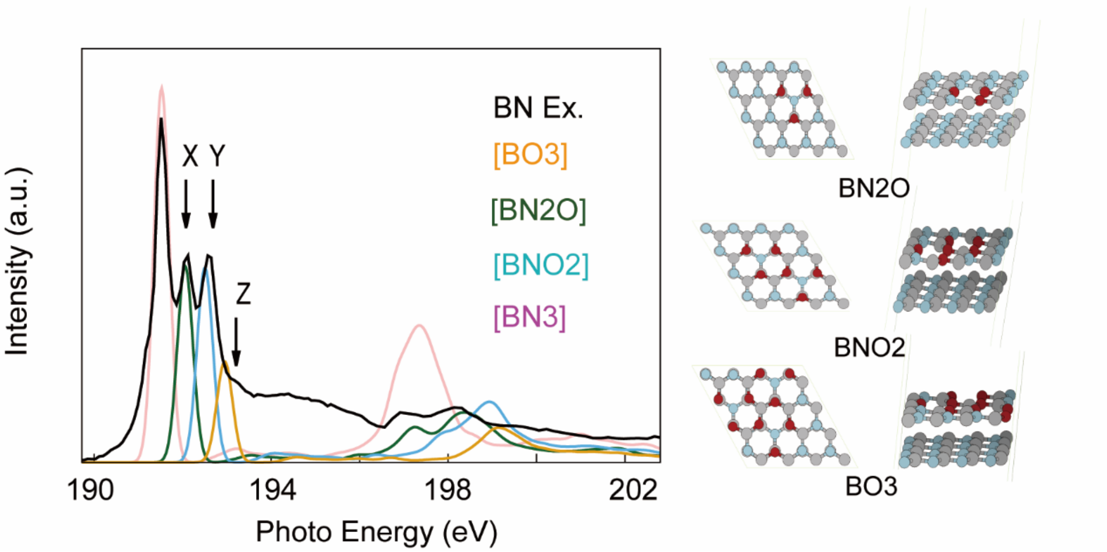

Fig. S11. Calculated B K-edge XAS spectra of O-terminated BN_3 vacancies on the hBN slabs (dotted lines), together with experimental bulk and exfoliated hBN XAS spectra. On the right panel, top and side views of the defected 4x4x1 hBN supercells used to model BN_2O, BNO_2 and BO_3 defects.

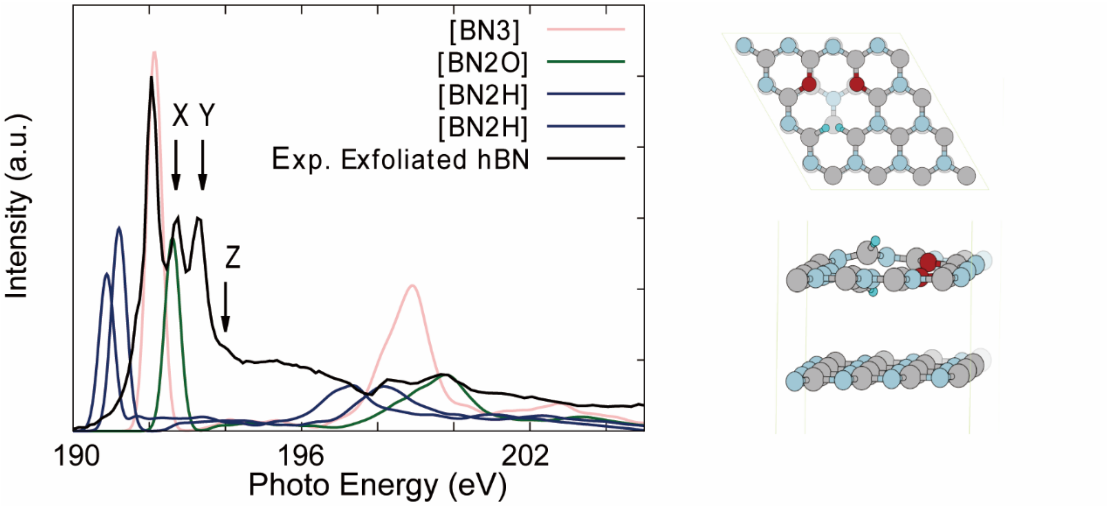

Fig.S12. Calculated B K-edge XAS spectra of O_2H_2 (BN_2O, BN_2H) -terminated BN_3 vacancies on the hBN slabs, together with experimental exfoliated hBN XAS spectra (dotted lines). On the right

panel, top and side views of the defected 4x4x1 hBN structure with an O_2H_2-terminated BN_3 vacancy.

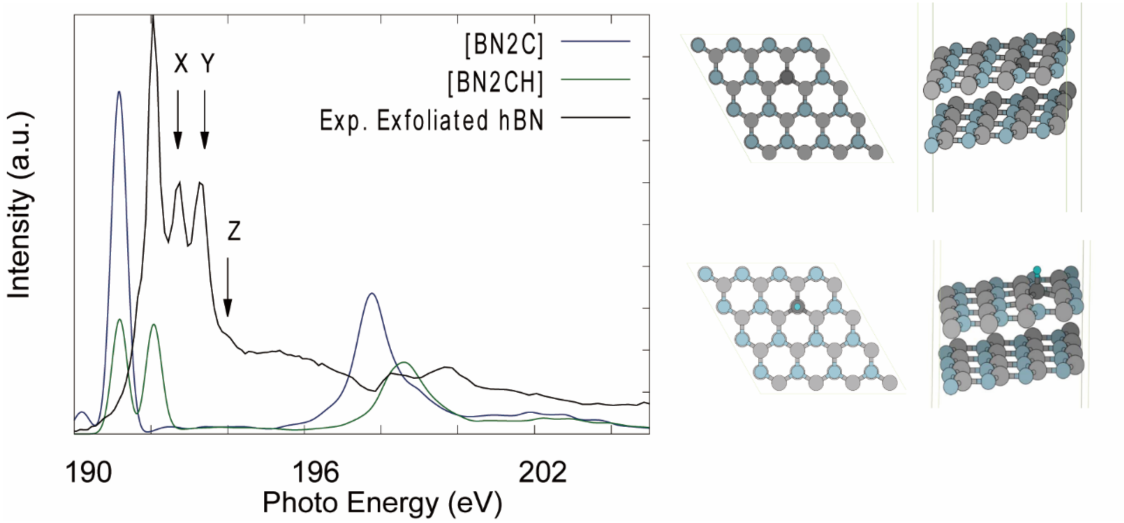

Fig. S13. Calculated B K-edge XAS spectra of C and C,H -terminated N vacancies on the hBN slabs, together with experimental exfoliated hBN XAS spectra (solid lines). On the right panel, top and side views of the defected 4x4x1 hBN structures with C and C,H impurities.

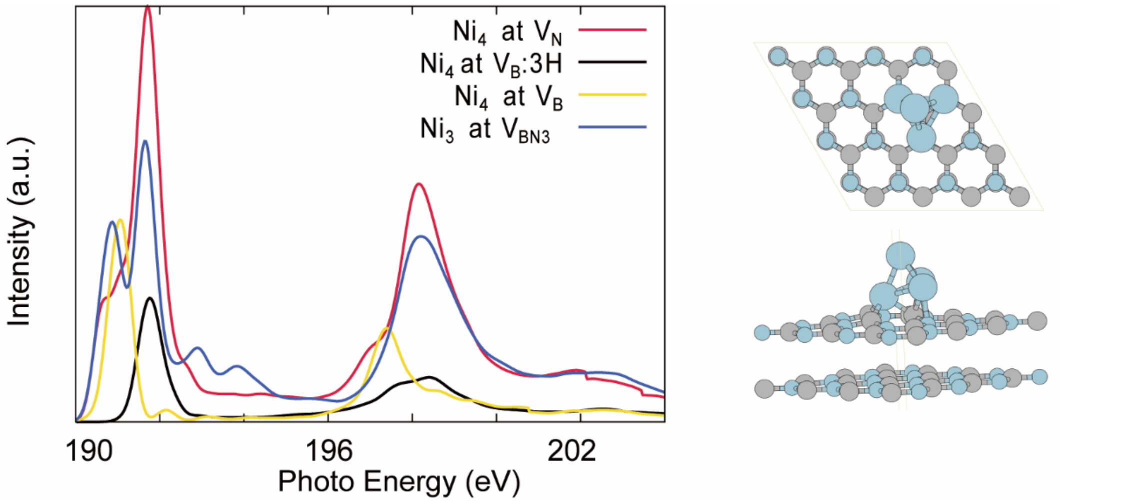

Fig. S14. Calculated B K-edge XAS spectra of Ni and Ni, H terminated defects. In the inset, top view of a Ni_4-terminated N_v.

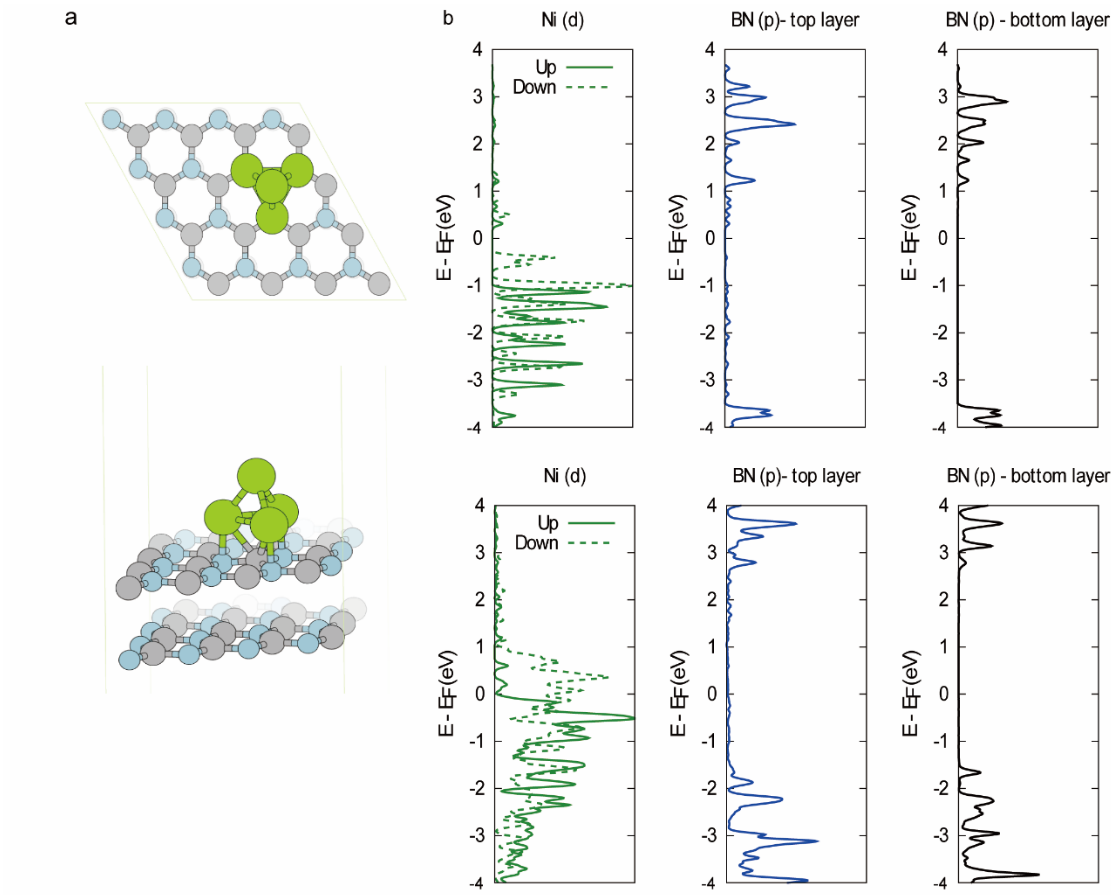

Fig. S15. a. Top and side views of the the structure of Ni_4 adsorbed on pristine hBN.

B. Projected density of states in Ni_4 (top) and Ni_{38} (bottom) corresponding to the Ni *d* orbitals (left), top-layer BN *p* orbitals (center) and bottom layer BN *p* orbitals (right).

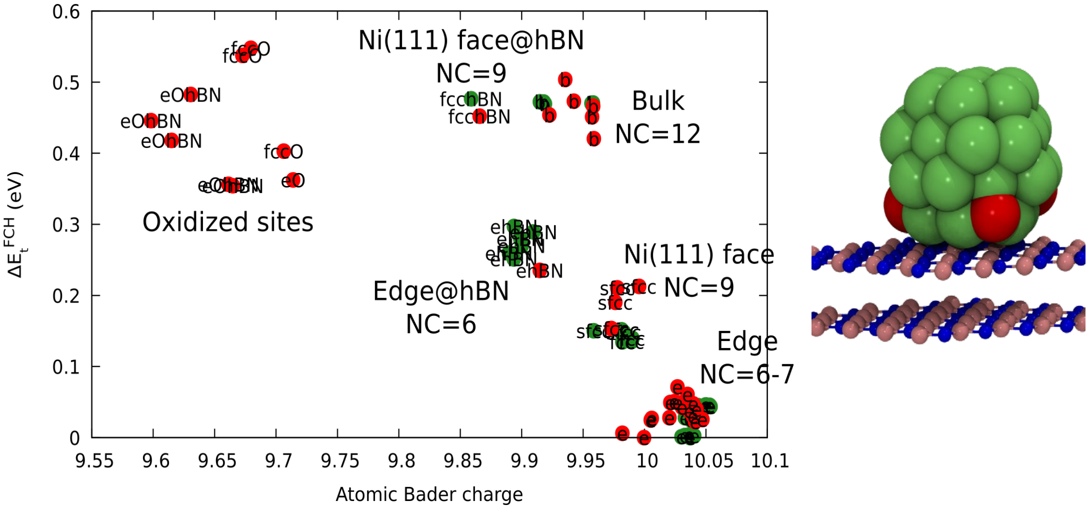

Fig. S16. Full core-hole excitation energies (left) of different Ni atoms in the pristine and lightly oxidized Ni_{38} (green) and $Ni_{38}O_3$ (red) clusters adsorbed on an hBN bilayer. The structure of $Ni_{38}O_3$ /hBN is shown on the right. The edge, face, and bulk sites are marked as e, f, and b.

Table S3. Estimated surface concentration C (V_{BN3}/nm^2) of O-terminated V_{BN3} defects (containing 6 [BN_2O] moieties) at the hBN surface, if a sample slab of one, three or nine hBN layers is considered, base on the relative intensity of the calculated and experimental W and X peaks The corresponding thickness is indicated in parenthesis. These can be considered an upper boundary.

		C		
Sample	Exp. X/W peak ratio	1 L (0.7 nm)	3 L (1.0 nm)	9 L (3.0 nm)
Bulk hBN	0.12	0.33	0.11	0.01
Exfoliated hBN	0.57	1.05	0.35	0.04
Ni/hBN	0.22	0.53	0.18	0.02

Section 3. Nickel nucleation on hBN

Table S4. Total magnetization (μB), adsorption energies (eV) and Ni-atom distances (Å) for single Ni atom adsorption on defected and pristine hBN sites, as calculated with PBE-D3+U (U=6.4 eV).

Site		E_{ads} (eV)	$d_{Ni\text{-}atom}$ (Å)
B (top)	2	−0.47	2.47
N (top)	0	0.40	1.87
BN_2O (top O)	0	0.85	1.88
BN_2O (center)	2	−0.50	2.57
BN_2O (top B)	2	−0.44	2.43

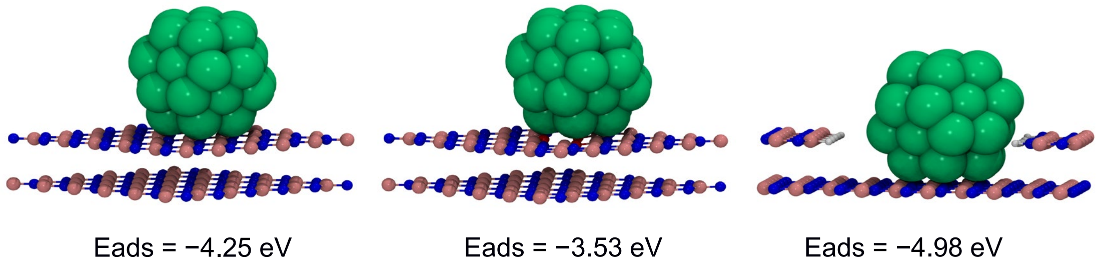

Fig. S17. Optimized structures and adsorption energies of Ni_{38} adsorbed on pristine hBN (left), O-terminated BN3 vacancy (center) and pristine hBN "pit".

Section 4. Methanol dehydrogenation and methanol formation

The adsorption energies of possible methanol dehydrogenation and methane formation were calculated as per the computational methods in Section S1. The mechanism of dehydrogenation at the Ni(111) surface has been thoroughly studied in the literature. Those are the steps used here (Table S5 and S6).

Table S5. Adsorption energies E_{ads} (eV) of species involved in methanol dehydrogenation and CO methanation on Ni_{38} and Ni(111). Note that they may be larger than reported in the literature because of the dispersion correction which has been included in these calculations.

Species	Ni(111)	Ni_{38}
CH_3OH	−0.65	−0.81
CH_3O	−2.74	−3.10
CH_2O	−0.99	−1.66
CHO	−2.43	−3.14
CO	−2.06	−2.18

Table S6. Relative energies of methanol dehydrogenation with respect to $CH_3OH_{(g)}$ on Ni(111) and Ni_{38}.

Species	Ni(111)	Ni_{38}
CH_3OH	0	0
CH_3OH*	−0.65	−0.81
CH_3O* +H*	−1.11	−1.43
CH_2O* + 2H*	−1.03	−1.62

$CHO^* + 3H^*$	−1.49	−2.09
$CO^* + 4H^*$	−2.87	−2.83

Table S7. C-O dissociation energies (eV) on the methanol dehydrogenation intermediates on Ni (111) and Ni_{38}.

	Ni(111)	Ni_{38}
$CH_3OH^* \rightarrow CH_3^* + OH^*$	−0.56	−0.99
$CH_3O^* \rightarrow CH_3^* + O^*$	−0.36	−0.42
$CH_2O^* \rightarrow$ CH2* + O*	−0.44	−0.59
$CHO^* \rightarrow CH^* + O^*$	−0.37	−0.67

We have considered the formation of methane under the assumption that all surface hydrogen is generated from methanol, according to the following reaction:

$$2\ CH_3OH \rightarrow CH_4 + CO + H_2O + H_2$$

where one molecule of methanol dehydrogenates as described in Fig. 4 of the main text, and the other molecule of methanol undergoes dissociative chemisorption followed by hydrogenation to CH_4, as shown in Figure S18. We have also included full dehydrogenation to C_f in order to provide a more complete energy landscape, despite this not being strictly a catalytic reaction since the C_f remains on the surface.

As shown, formation of CO* and H* is the most favored final state, yet some steps involving CH_X species are more exothermic. It should be noted that the energetics shown here offer a slightly limited picture, since the reaction network may be more extense (e.g. including minoritary HCOH aspecies). The lack of activation energies precludes from making kinetic considerations.

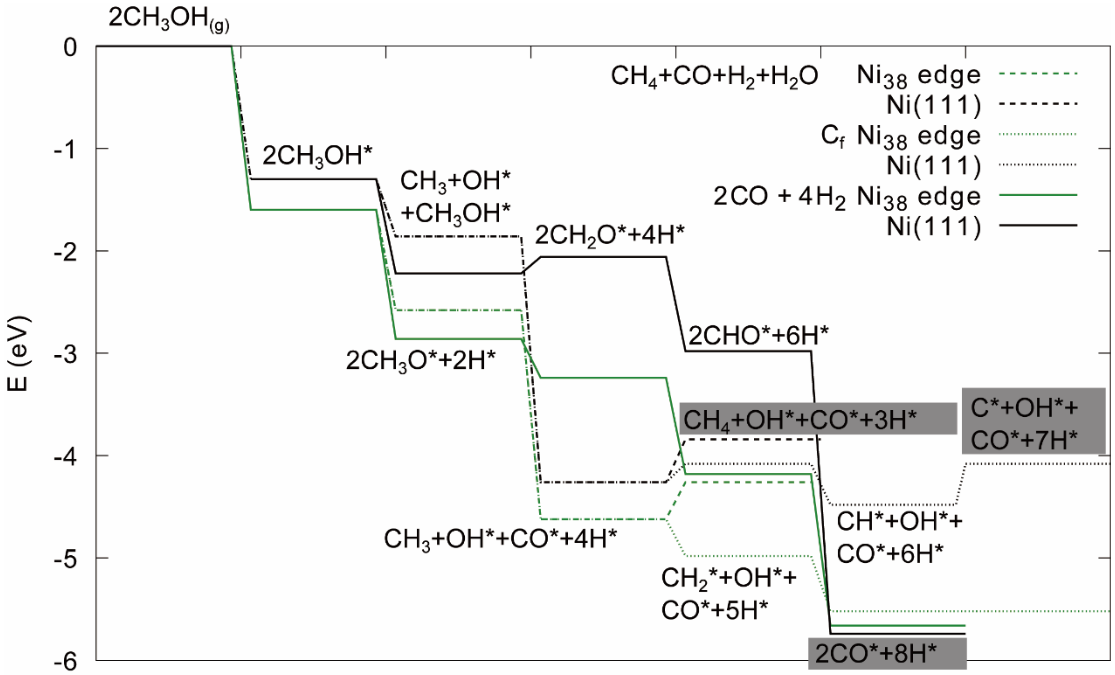

Fig. S18. Relative energies of the intermediates of the methanol dehydrogenation reaction, together with those leading to CH_4 and C_f formation. The lines between states are meant to guide the eye.

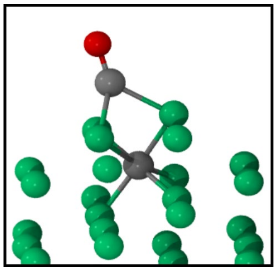

Fig. S19. Atomic structure of CO adsorbed on the Ni38 subsurface when a subsurface carbon atom is present.

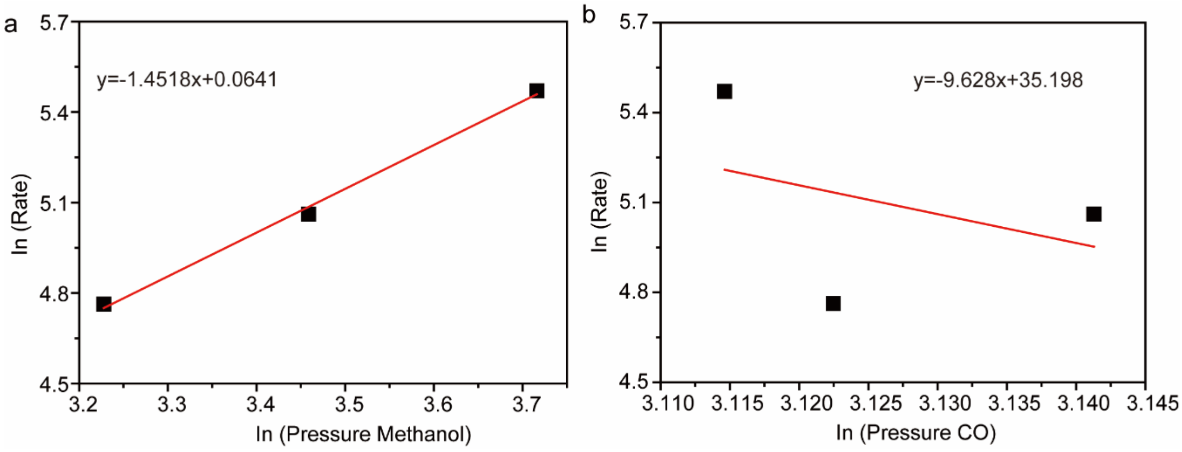

Figure S20. The reaction kinetic of methanol decomposition on Ni-BN catalyst.

Kinetic measurements of the specific reaction rates we are testing were usually performed in a low conversion regime (such as15%), to ensure that concentration changes do not affect the observed reaction rate. Furthermore, for specific methanol decomposition, since carbon monoxide and hydrogen significantly affect the reaction rate, the CO and H_2 gases were often co-fed in order to change the reaction composition. For our catalytic data presented in the inset of Figure 5b, the conversion results are arranging from 55 % to 100 %. Methanol decomposition reaction is a volume increasing reaction (as one methanol generate one CO and two H_2). The volumetric change at high conversion of methanol will generate obvious concentration changes which can substantially affect the reaction rate. Therefore, the kinetic calculations based on these high conversion data contain obvious sources of potential error. However, we calculated the reaction order of our methanol decomposition based on catalytic date presented on Figure 5 b. As shown in the above figures, the reaction order of methanol decomposition on Ni-BN catalyst is about 1.45. Unfortunately, the Ln(rate) and Ln(Pressure CO) is not a linear relationship. These complications meant that the data collected could not be used to provide straightforward mechanistic insights.

Reference

1. J. C. Brown & E. Gulari, Hydrogen production from methanol decomposition over Pt/Al_2O_3 and ceria promoted Pt/Al_2O_3 catalysts. *Catal. Commun.* **5**, 431-436 (2004).
2. G. Avgouropoulos, J. Papavasiliou, & T. Ioannides, Hydrogen production from methanol over combustion-synthesized noble metal/ceria catalysts. *Chem. Eng. J.* **154**, 274-280 (2009).
3. T. Tsoncheva*, et al.,* Nickel modified ultrananosized diamonds and their application as catalysts in methanol decomposition. *J. Mol. Catal. A-Chem.* **259**, 223-230 (2006).
4. S. D. Lin, T. C. Hsiao, & L. C. Chen, The steady state methanol decomposition reaction over Cu/Zn and Cu/Cr catalysts: Pretreatment, operando EXAFS, and activity study. *Appl. Catal. A-Gen.* **360**, 226-231 (2009).
5. T. Tsoncheva, L. Ivanova, C. Minchev, & M. Fröba, Cobalt-modified mesoporous MgO, ZrO_2, and CeO_2 oxides as catalysts for methanol decomposition. *J. Colloid Interface Sci* **333**, 277-284 (2009).
6. Z. H. Xue*, et al.*, Tuning the Adsorption Energy of Methanol Molecules Along Ni-N-Doped Carbon Phase Boundaries by the Mott–Schottky Effect for Gas-Phase Methanol Dehydrogenation. *Angew. Chem. Int. Ed.* **130**, 2727-2731(2018).
7. L. Weston, D. Wickramaratne, M. Mackoit, A. Alkauskas, & C. Van de Walle, Native point defects and impurities in hexagonal boron nitride. *Phys. Rev. B* **97**, 214104 (2018).
